# Supplementary material for: Restoration of functional PAX6 in aniridia patient iPSC-derived ocular tissue models using repurposed nonsense suppression drugs
Source: Mol Ther Nucleic Acids. 2023 Jun 26;33:240–53. doi: 10.1016/j.omtn.2023.06.016 (PMC10362734; doi:10.1016/j.omtn.2023.06.016)
Supplement: Document S1. Figures S1–S6 and Table S1 [file mmc1.pdf]

OMTN, Volume *33*

Supplemental information

Restoration of functional PAX6 in aniridia patient iPSC-derived ocular tissue models using repurposed nonsense suppression drugs

Dulce Lima Cunha, Hajrah Sarkar, Jonathan Eintracht, Philippa Harding, Jo Huiqing Zhou, and Mariya Moosajee

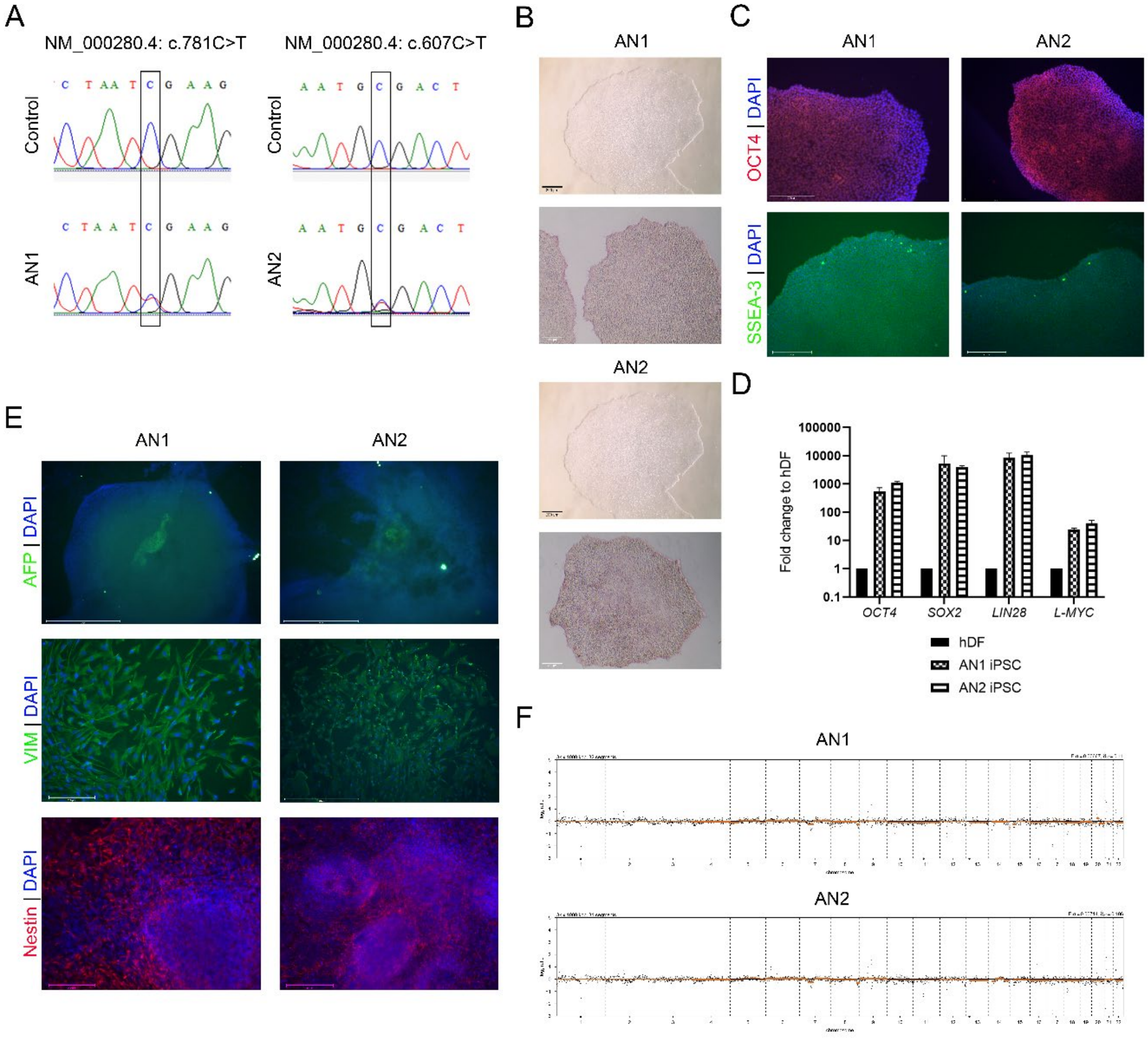

Figure S1. Characterisation of iPSCs generated from two independent aniridia patient (AN1 and AN2) carrying *PAX6* heterozygous nonsense variants. (A) The heterozygous nonsense variants c.781C>T, p.Arg261* (AN1) and c.607C>T, p.(Arg203*) (AN2) in the *PAX6* gene (NM_000280.4) were confirmed in each aniridia iPSC line through direct sequencing and were absent from control iPSCs. (B) Embryonic stem cell-like morphology and positive alkaline phosphatase (red) staining. (C) Positive expression of pluripotency markers OCT4 (upper panel) and SSEA-3 (lower panel). Scale bar 200µm. (D) Pluripotency marker genes *OCT4*, *SOX2*, *L-MYC* and *LIN28* were upregulated in both AN iPSC compared to each parental fibroblasts (hDF) line by qRT-PCR. (E) In vitro differentiation ability was confirmed by random differentiation of both aniridia iPSCs: cells

stained positive for endoderm (AFP), mesoderm (Vimentin) and ectoderm (Nestin) markers. (F) Low-pass whole genome sequencing analysis revealed no abnormalities in AN1 and AN2 iPSCs, showing 46,XY karyotype in both cases.

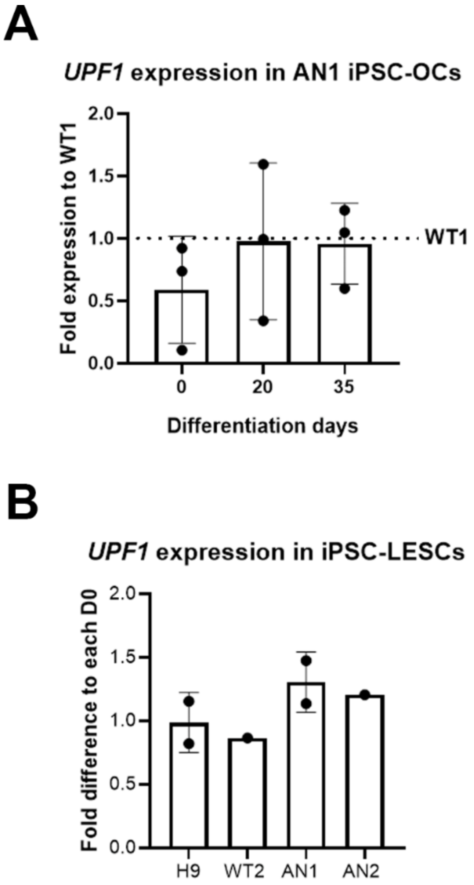

Figure S2. NMD status in iPSC-derived models. (A) *UPF1* mRNA expression levels in AN1 iPSC-OCs were measured by qRT-PCR on differentiation days 0, 20 and 35 and normalised to WT1 expression on the same day (dotted line). No significant changes were detected (n=3). (B) *UPF1* mRNA expression in control (H9 and WT2) and aniridia (AN1 and AN2) iPSC-LESCs on differentiation day 15 and normalised to each line's day 0.

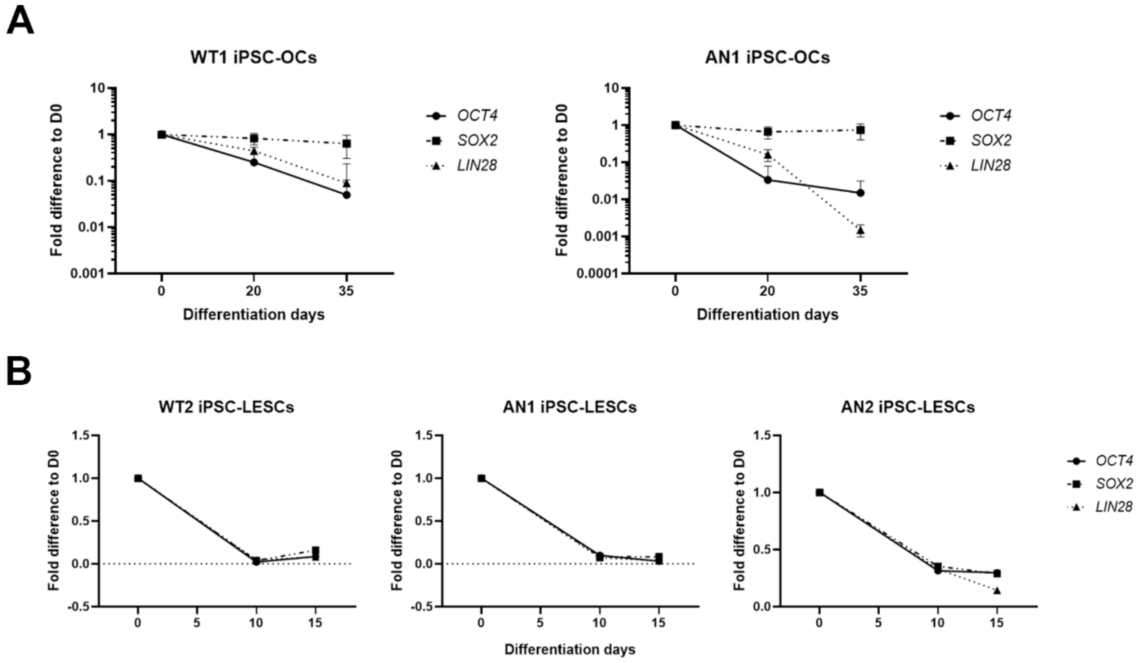

Figure S3. Pluripotency genes expression during WT and AN differentiation. (A) Expression of *OCT4*, *SOX2* and *LIN28* was detected by qRT-PCR on days 0, 20 and 35 of optic cup (OCs) differentiation. *OCT4* and *LIN28* were significantly downregulated; SOX2 is required for early eye development, hence no mRNA reduction is seen compared to day 0. (B) All three markers were downregulated during iPSC differentiation into limbal epithelial stem cells (LESCs), for all lines tested. Values were normalised to day 0 and to internal housekeeper gene *GAPDH.*

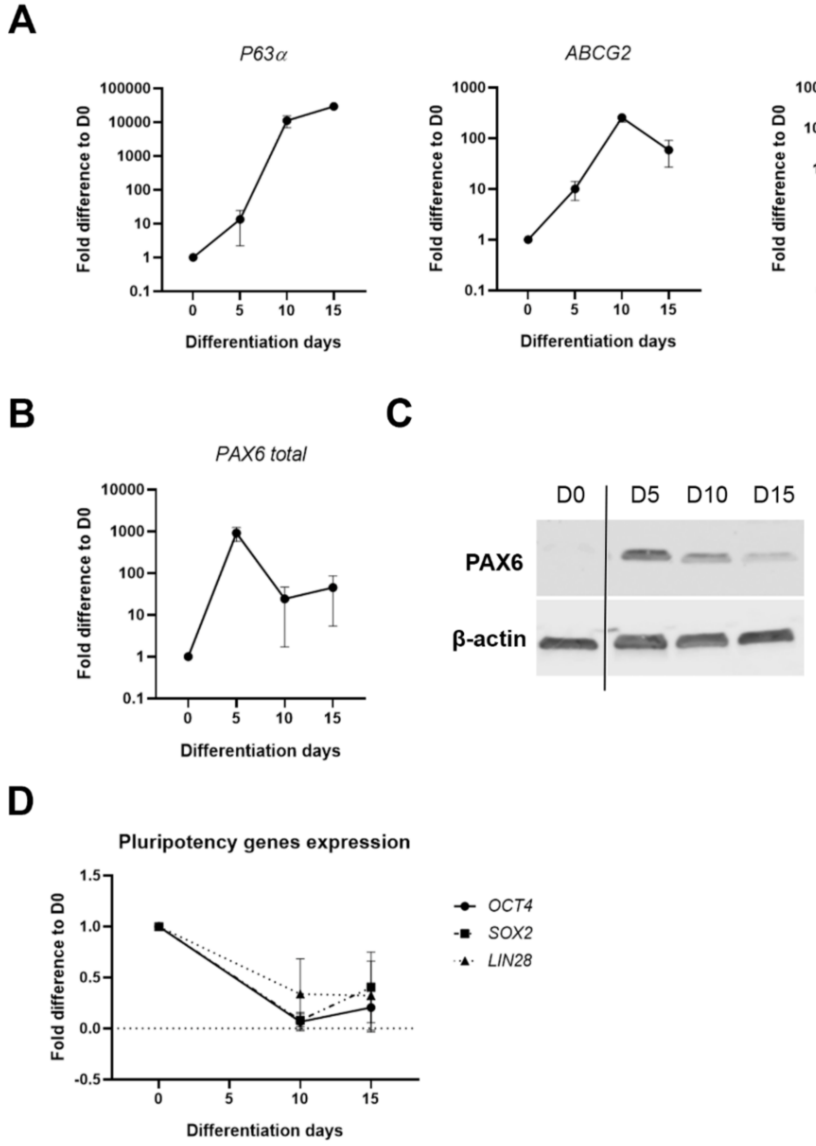

Figure S4. H9 embryonic stem cell (ESC) differentiation into Limbal epithelial stem cells (LESCs). (A) Expression of LESC specific markers *P63α, ABCG2* and *KRT14* was upregulated by day 15 and comparable to other lines shown in Figure 2. (B) The same pattern of expression of *PAX6* was also shown in this cell line. Values were normalised to day 0 and to internal housekeeper gene *GAPDH* (n=2). (C) Example of western blot showing PAX6 protein levels at different timepoints of LESC differentiation. (D) Pluripotency markers *OCT4*, *SOX2* and *LIN28* were downregulated through differentiation (n=2).

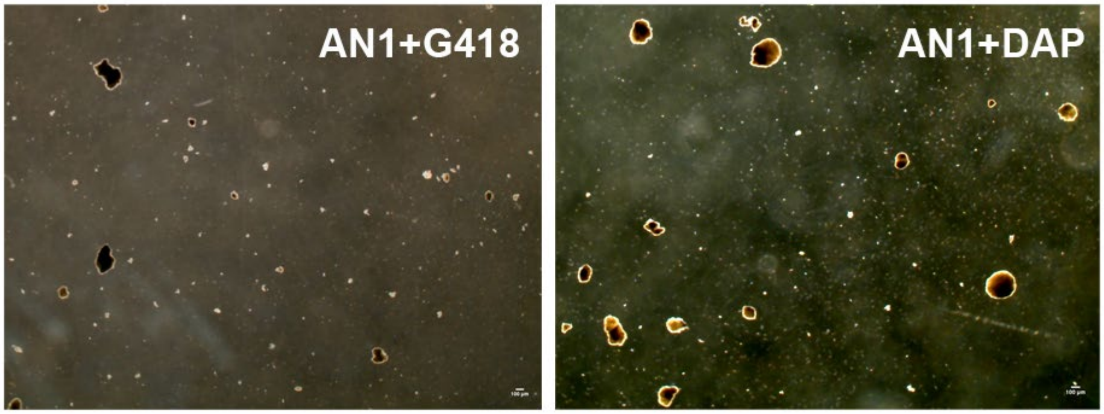

Figure S5. Cell toxicity after treatment of aniridia iPSC-derived optic cups with G418 100µg/mL and 2,6-diaminopurine (DAP) 200µM. Cell clumps progressively darkened after starting of dosing (day 15) and no viable structures were seen after day 20 (G418) or 25 (DAP) of treatment. Scale bar, 100µm.

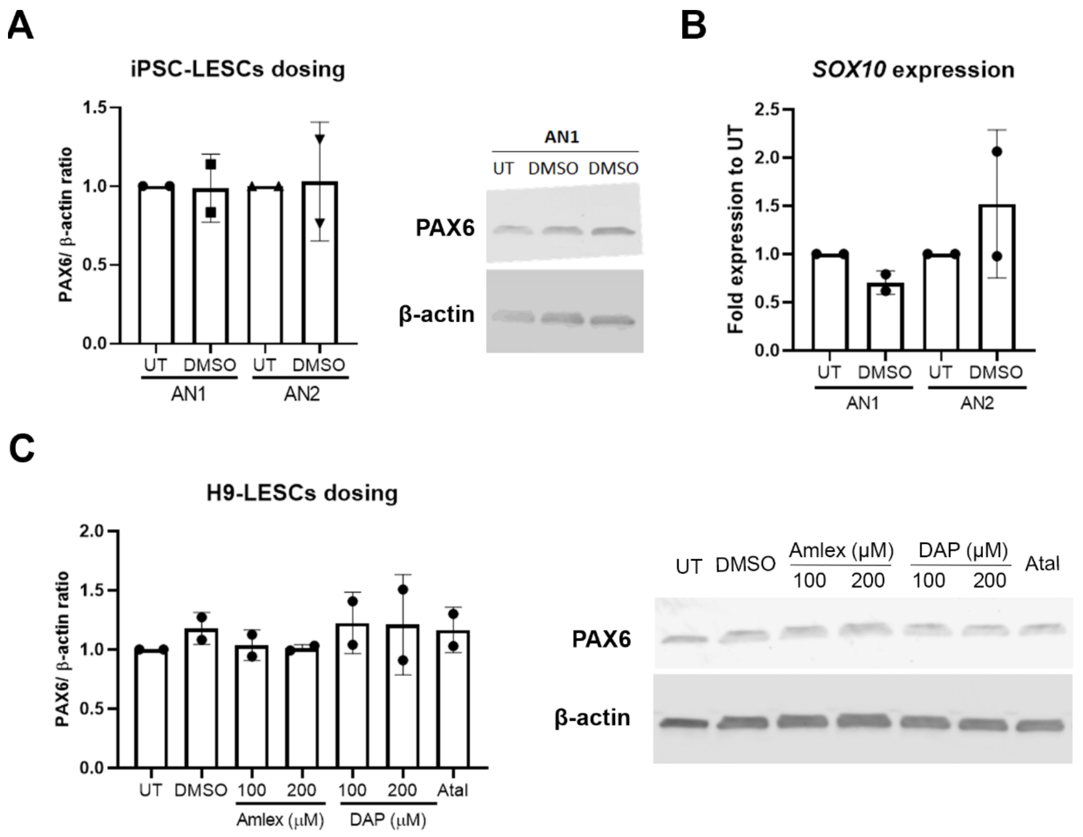

Figure S6. TRIDs dosing of aniridia and control (H9) iPSC-LESCs. (A) No significant changes found in PAX6 protein levels in AN1 and AN2 iPSC-LESCs untreated (UT) or after 48h dosing with vehicle (DMSO) treatment (n=2). (B) *SOX10* expression detected by qRT-PCR was not significantly different between UT and DMSO-treated AN1 and AN2 iPSC-LESCs (n=2). (C) Quantification of PAX6 protein in control H9 ESC-derived LESCs treated with different TRIDs. PAX6/β-actin ratio was normalised to untreated H9-LESCs. No significant differences between the different conditions were detected (n=2).

Table S1. Primer sequences used for qRT-PCR.

Marker	Forward sequence (5’-3’)	Reverse sequence (5’-3’)	Reference
GAPDH	ACA GTT GCC ATG TAG ACC	TTT TTG GTT GAG CAC AGG	In house
ACTB	TTC TAC AAT GAG CTG CGT G	GGG GTG TTG AAG GTC TCA AA	In house
PAX6	GGC CGA ACA GAC ACA GCC CTC AC	ATC ATA ACT CCG CCC ATT CAC C	In house
RAX	AGG CGG AAA AAT AGA GTT TG	TAC CCC AAT ATT CAC TCC TC	KickStart, Sigma Aldrich
VSX2	GGC GAC ACA GGA CAA TCT TTA	TTC CGG CAG CTC CGT TTT C	KickStart, Sigma Aldrich
MKi67	AAA CCA ACA AAG AGG AAC ACA AAT T	GTC TGG AGC GCA GGG ATA TTC	In house
TP63α	ATG TCG AAA TTG CTC AGG GAT TTT CAG A	TGA CCA CCA TCT ATC AGA TTG AGC ATT ACT	Foster et al, 2019
ΔNP63	GAA AAC AAT GCC CAG ACT CAA TTT	TCT GCG CGT GGT CTG TGT TAT	Foster et al, 2019
ABCG2	TCC ACT GCT GTG GCA TTA AA	CCT GCT TGG AAG GCT CTA TG	Foster et al, 2019
KRT14	CGG CCT GCT GAG ATC AAA GA	TCT GCA GAA GGA CAT TGG CA	Foster et al, 2019

SOX10	CTC TGG AGG CTG CTG AA	TGG GCT GGT ACT TGT AGT C	Leung et al, 2016
