# Supplementary material for: Restoration of functional PAX6 in aniridia patient iPSC-derived ocular tissue models using repurposed nonsense suppression drugs
Source: Mol Ther Nucleic Acids. 2023 Jun 26;33:240–53. doi: 10.1016/j.omtn.2023.06.016 (PMC10362734; doi:10.1016/j.omtn.2023.06.016)
Supplement: Document S2. Article plus supplemental information [file mmc2.pdf]

Molecular Therapy
Nucleic Acids
Original Article

Restoration of functional PAX6 in aniridia patient iPSC-derived ocular tissue models using repurposed nonsense suppression drugs

Dulce Lima Cunha,[1,2] Hajrah Sarkar,[1,3] Jonathan Eintracht,[1] Philippa Harding,[1] Jo Huiqing Zhou,[2] and Mariya Moosajee[1,3,4]

[1]UCL Institute of Ophthalmology, London, UK; [2]Radboud Institute of Molecular Life Sciences, Radboud University, Nijmegen, the Netherlands; [3]The Francis Crick Institute, London, UK; [4]Moorfields Eye Hospital, London, UK

Congenital aniridia is a rare, pan-ocular disease causing severe sight loss, with only symptomatic intervention offered to patients. Approximately 40% of aniridia patients present with heterozygous nonsense variants in *PAX6*, resulting in haploinsufficiency. Translational readthrough-inducing drugs (TRIDs) have the ability to weaken the recognition of in-frame premature termination codons (PTCs), permitting full-length protein to be translated. We established induced pluripotent stem cell (iPSC)-derived 3D optic cups and 2D limbal epithelial stem cell (LESC) models from two aniridia patients with prevalent *PAX6* nonsense mutations. Both *in vitro* models show reduced PAX6 protein levels, mimicking the disease. The repurposed TRIDs amlexanox and 2,6-diaminopurine (DAP) and the positive control compounds ataluren and G418 were tested for their efficiency. Amlexanox was identified as the most promising TRID, increasing full-length PAX6 levels in both models and rescuing the disease phenotype through normalization of VSX2 and cell proliferation in the optic cups and reduction of ABCG2 protein and *SOX10* expression in LESCs. This study highlights the significance of patient iPSC-derived cells as a new model system for aniridia and proposes amlexanox as a new putative treatment for nonsense-mediated aniridia.

INTRODUCTION

Aniridia (MIM: 106210) is a rare, dominant, pan-ocular disease with a prevalence of 1 in 40,000–100,000.[1] Typical symptoms of this disease include congenital iris and foveal hypoplasia with nystagmus and progressive development of glaucoma, cataracts, and keratopathy, leading to significant visual impairment.[2–4] Up to 90% of aniridia patients develop limbal stem cell deficiency (LSCD), where adult epithelial stem cells originating in the limbus and maintaining corneal transparency, are lost or defective, causing impaired epithelium renewal and conjunctival invasion.[5] LSCD invariably results in complete corneal opacity, usually termed aniridia-related keratopathy (ARK), and is the most relevant feature contributing to visual loss in aniridia post-natally.[5,6]

Heterozygous mutations affecting the *PAX6* gene or its regulatory regions are the cause of aniridia,[7,8] with mutations introducing a premature termination codon (PTC) being the most common (http://lsdb.hgu.mrc.ac.uk/home.php?select_db=PAX6). Of these, nonsense mutations are the most prevalent, accounting for 39% of the total mutations reported in aniridia patients.[9] *PAX6* nonsense mutations are predicted to result in loss of function, where mutated mRNA is likely degraded by nonsense-mediated decay (NMD), resulting in *PAX6* haploinsufficiency.

Nonsense suppression or translational readthrough-inducing drugs (TRIDs) weaken recognition of a PTC and promote replacement of a near-cognate amino acid, thus allowing translation to continue and producing a full-length protein.[10,11] Promising preclinical data using ataluren (also called Translarna or PTC124), a TRID approved for treatment of Duchenne muscular atrophy, showed rescue of Pax6 levels in the aniridia *Sey*$^{+/-}$ mouse model, with topical administration inhibiting disease progression and improving corneal, lens, and retinal defects.[12,13] A phase I/II clinical trial (ClinicalTrials.gov: NCT02647359) for aniridia was completed but failed to meet the primary endpoint despite showing a positive trend towards functional improvement (https://www.prnewswire.com/news-releases/ptc-therapeutics-reports-fourth-quarter-and-full-year-2019-financial-results-and-provides-a-corporate-update-301014669.html). The use of TRIDs is a particularly suitable therapeutic approach for aniridia because of the high prevalence of nonsense variants and the milder phenotype associated with *PAX6* missense mutations.[2,3,9] However, novel readthrough compounds with improved efficiency are required with a personalized medicine approach, knowing which TRIDs may be more effective for specific PTCs or that combined inhibition of NMD may boost mRNA substrate and end protein production.

PAX6 is a dose-sensitive transcription factor essential for eye development.[7,8] It is expressed early in ocular morphogenesis, during

Received 26 May 2022; accepted 22 June 2023;
https://doi.org/10.1016/j.omtn.2023.06.016.

Correspondence: Mariya Moosajee, PhD, UCL Institute of Ophthalmology, 11-43 Bath Street, EC1V 9EL London, UK.
E-mail: m.moosajee@ucl.ac.uk

 © 2023 The Author(s).
This is an open access article under the CC BY license (http://creativecommons.org/licenses/by/4.0/).

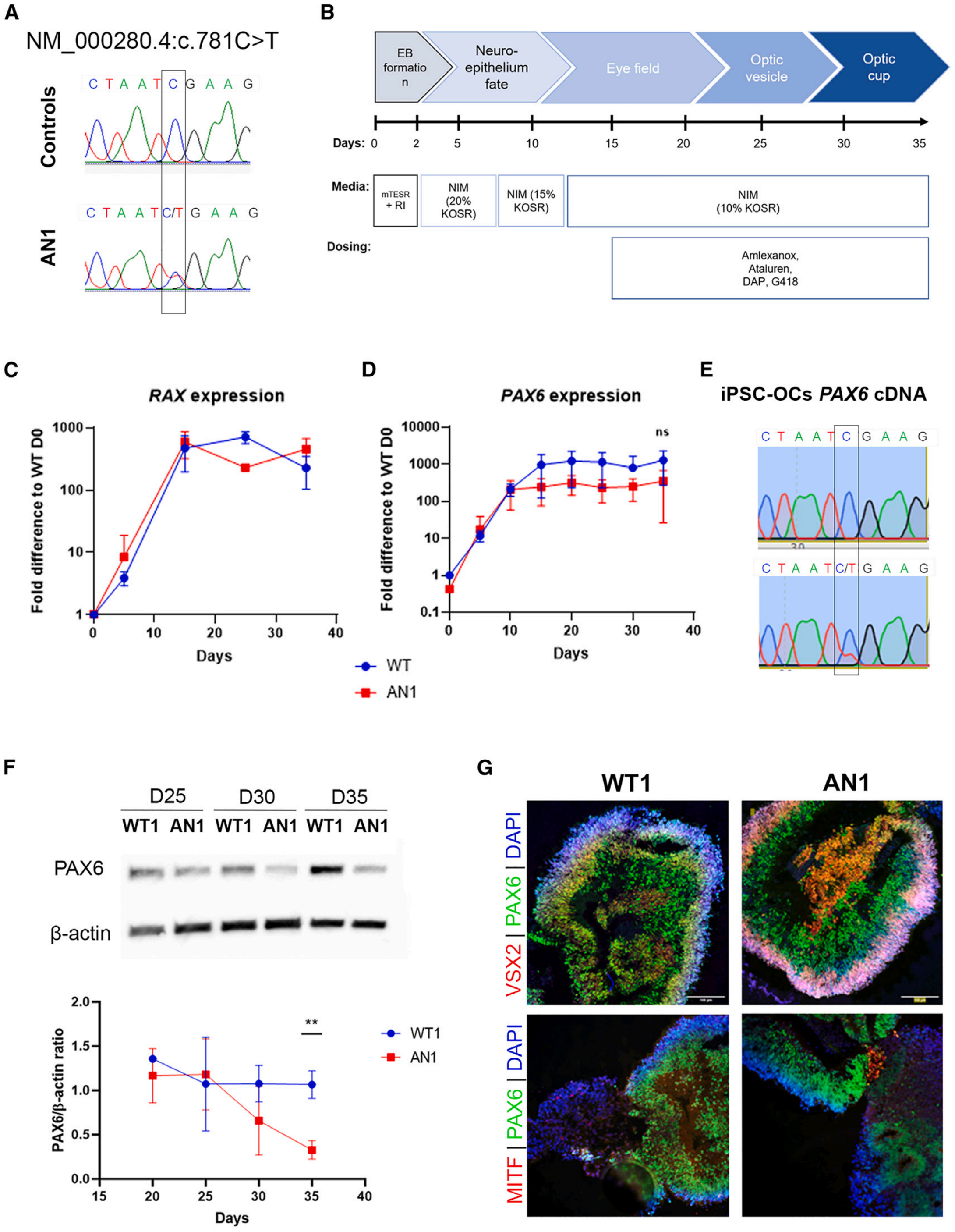

Figure 1. Generation of AN iPSC-derived OCs

(A) Direct sequencing of *PAX6* exon 10, showing the heterozygous nonsense c.781C>T change in AN1 patient iPSCs. This variant was not detected in control lines. (B) Schematic of the differentiation strategy of control WT1 and WT2 and patient-derived AN1 iPSCs into 3D OCs (35 days). Data from the WT1 control line are included as an example. Dosing experiments with TRIDs were performed from day 15 onwards. (C and D) qRT-PCR transcript analysis of the EFTFs *RAX* and *PAX6* during 35 days of differentiation in control (WT1, blue) and aniridia (AN1, red) iPSCs. Values were normalized to day 0 and to the internal housekeeping gene *GAPDH*. Data represents means and SD of 3 biological replicates. (E) RT-PCR followed by Sanger sequencing of *PAX6* cDNA from day 35 WT1 and AN1 iPSC-OCs. The mutated allele c.781C>T can be seen

(legend continued on next page)

establishment of the eye field and optic vesicle, and has multiple roles in the development and maintenance of retinal progenitor cells, lens, cornea, and iris.[14] In the cornea, correct Pax6 levels are required for normal cell growth during limbal and central corneal epithelial development, but the exact mechanisms of how *PAX6* haploinsufficiency causes LSCD and ARK are still not understood.[15] It has been shown recently that Pax6 controls neural crest migration during corneal development, a process important for formation of the non-epithelial corneal layers (i.e., stroma and endothelium) as well as for maintenance of the limbal niche.[16–18]

Generation of human induced pluripotent stem cells (iPSCs) has opened a new avenue in establishing representative *in vitro* models that can recapitulate human development and provide valuable insights into disease mechanisms.[19] They have been used to accelerate therapeutic development in several retinal and corneal eye disorders.[20–22] This is the first study to generate iPSCs from aniridia patients carrying heterozygous *PAX6* nonsense mutations with a UGA-type PTC and to establish patient-specific iPSC-derived optic cups and limbal epithelial stem cell (LESC) models that mimic the haploinsufficiency state. We used these models to assess the potential of the TRIDs amlexanox and 2,6-diaminopurine (DAP) to treat aniridia. Amlexanox is a US Food and Drug Administration (FDA)-approved drug used for treatment of asthma and aphthous mouth ulcers,[23] that was found to have readthrough and NMD inhibition properties.[24–26] DAP is an antileukemia compound with recently identified strong readthrough capacity for UGA-type PTC.[27]

We identified amlexanox as the most promising TRID, increasing full-length PAX6 levels and rescuing phenotype abnormalities in iPSC-derived retinal and corneal models, while DAP showed distinct tissue-dependent responses. Our results provide a substantial proof of concept for use of amlexanox as a new therapeutic approach for aniridia.

RESULTS

Generation of aniridia iPSCs

Human dermal fibroblasts taken from two molecularly confirmed aniridia patients (aniridia 1 [AN1] and 2 [AN2]) were reprogrammed into iPSCs by electroporation using non-integrating episomal plasmids.[28,29] Generated iPSC clonal lines were thoroughly and routinely characterized, showing positive pluripotency markers, tri-lineage differentiation ability, and chromosomal stability (Figure S1). AN1 carries a heterozygous nonsense variant in *PAX6* (NM_000280.4; c.781C>T/p.(Arg261*)), while AN2 carries the heterozygous nonsense variant c.607C>T/p.(Arg203*). Both variants are predicted to introduce a UGA PTC. The disease-causing variants were confirmed in each AN iPSCs by direct sequencing of *PAX6* exons 10 (AN1) and 8 (AN2) (Figures 1A and S1A).

AN iPSC-derived optic cups show reduced PAX6 protein but not mRNA levels

AN1 iPSCs as well as two independent iPSC lines derived from unaffected healthy controls (wild type 1 [WT1] and 2 [WT2]) were further differentiated into a 3D optic cup (OC)-like stage by adapting established protocols[30,31] (Figure 1B). Differentiating organoids showed upregulation of the eye field transcription factors (EFTFs) *RAX* and *PAX6* from day 10 onwards (Figures 1C and 1D).[32,33] No significant differences in *PAX6* mRNA levels were detected between AN1 and WT1 and WT1 iPSC OCs throughout the process, although downregulation compared with the WT seems apparent from day 15 (Figure 1D). RT-PCR of *PAX6* cDNA shows the presence of an AN1 mutated transcript (Figure 1E), while *UPF1* expression, a key activator of NMD, is unchanged compared with WT1 (Figure S2A), suggesting NMD escape. In contrast, PAX6 protein immunoblotting showed a significant reduction of PAX6 protein in AN iPSC-OCs at day 35, with approximately 0.33 ± 0.23-fold of WT1 and WT2 levels ($p < 0.01$; Figure 1F). Despite the reduced PAX6 protein, AN1 organoids could progress into an OC-like stage, typically around differentiation day 35, when the neural retina marker VSX2 (visual system homeobox 2) and retinal pigmented epithelium (RPE) marker MITF (microphthalmia-associated transcription factor) are present (Figure 1G). Expression of the pluripotency markers *OCT4* and *LIN28* was reduced throughout the differentiation process, showing exit from the pluripotency state (Figure S3A).

Establishment and characterization of AN iPSC-derived LESCs

To test the clinical potential of TRIDs as a possible therapy for AN LSCD, we differentiated the two AN iPSC lines (AN1 and AN2), together with the control lines WT1 and WT2, into 2D LESCs.[34] A third control, the H9 embryonic stem cell (ESC) line, was also included at this stage to limit the inter-donor variability often seen in iPSCs.[35] Following formation of embryoid bodies (EBs), limbal fate was induced for 5 days, and EBs were plated onto collagen IV-coated pates, where epithelial-like cells emerged and proliferated until day 15 (Figure 2A). Time point analysis confirmed high expression of the LESC-specific markers *ΔNP63α, KRT14*, and *ABCG2* by day 15 in all AN and WT control lines, proving limbal commitment (Figure 2B); this was also confirmed in the differentiated H9 ESC line (Figure S4). In parallel, the pluripotency markers *OCT4*, *SOX2*, and *LIN28* were downregulated for all lines, showing exit from the pluripotency state (Figure S3B).

Similar to our 3D OC model, no clear differences in *PAX6* mRNA expression were detected between our AN and WT lines (Figure 2C); however, a significant reduction in full-length PAX6 protein was seen

in AN1 but not in WT1 iPSC-OCs. (F) PAX6 protein analysis detected by western blot in WT and AN1 iPSC-OCs from days 25–35 of differentiation (5-day intervals). The PAX6/β-actin ratio was normalized to WT1. Data expressed as mean ± SD from n = 3 (**p < 0.01, t test analysis). (G) Immunohistochemical analysis of WT1 and AN iPSC-derived OCs, showing positive staining of PAX6 (green) as well as markers for OC domains: VSX2, indicating the neural retina (red, top panel), and MITF, indicating retinal pigmented epithelium (RPE; red, bottom panel). DAPI staining (blue) shows cell nuclei. Scale bar, 100 μm.

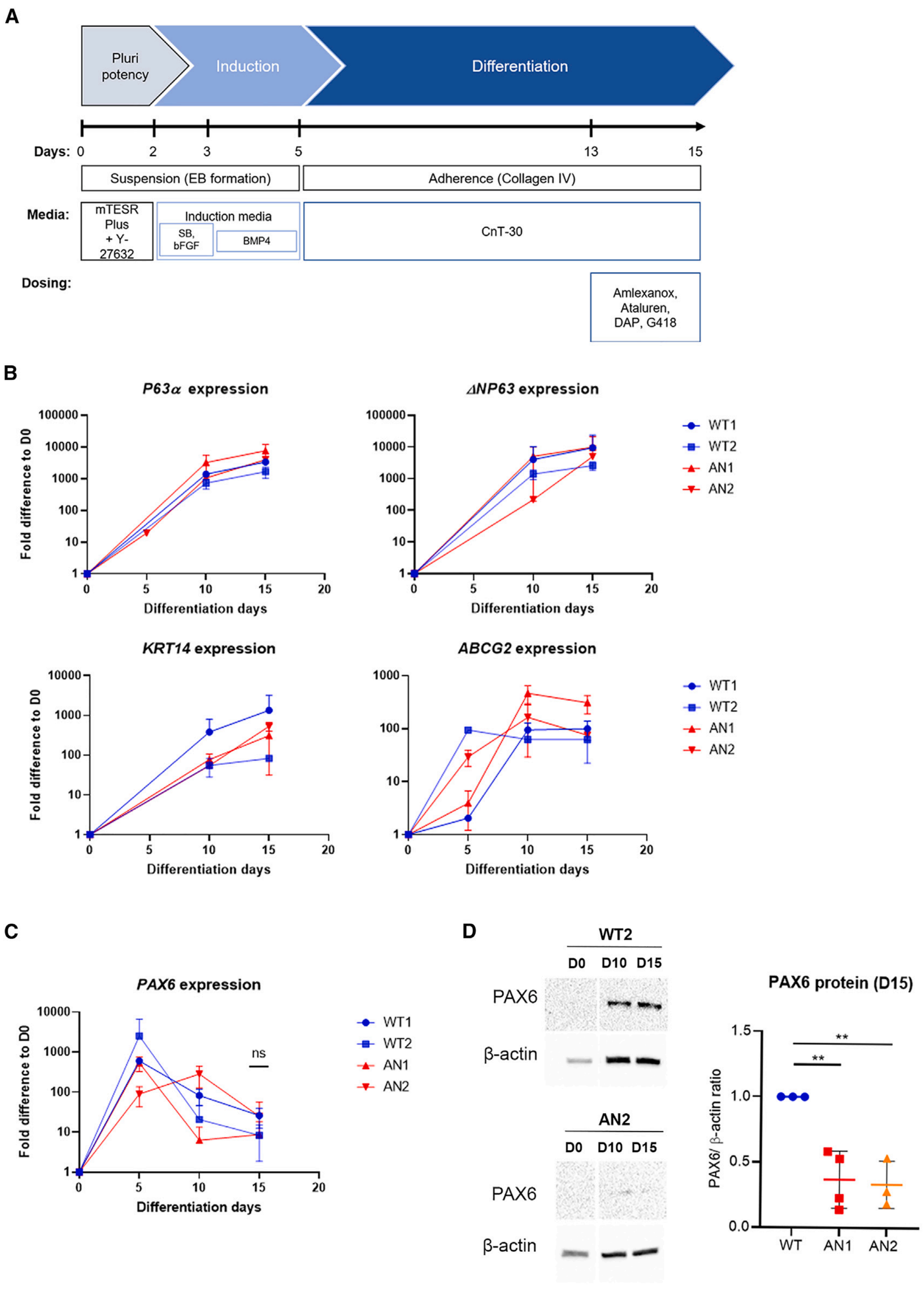

Figure 2. Characterization of iPSC-derived limbal epithelial stem cells (LESCs) from 2 AN patients

(A) Schematic of the differentiation protocol used in this study, based on Hongisto et al.[34] (B) qRT-PCR transcript analysis of the LESC markers *ΔNP63α* (measured with 2 primer pairs), *KRT14*, and *ABCG2* in 2 AN (AN1 and AN2) and 2 independent control (WT1 and WT2) iPSC lines, showing limbal commitment by day 15 of differentiation.

(legend continued on next page)

in AN1 and AN2 iPSC-LESCs after protein analysis on day 15, with both lines showing approximately one-third of PAX6 control levels (AN1: 0.37 ± 0.13 fold; AN2: 0.33 ± 0.14 fold vs. WT = 1, $p < 0.01$) (Figure 2D). NMD activity was assessed through *UPF1* expression, which remained unchanged between the different iPSC (and ESC)-derived LESCs (Figure S2B).

Amlexanox and ataluren increase full-length PAX6 levels in AN iPSC-OCs

To test the potential of TRIDs to increase full-length PAX6 levels, AN iPSC-OCs were dosed with the readthrough compounds amlexanox and DAP as well as ataluren and G418 from day 15 until collection on day 35 (Figure 1B). G418 caused cell toxicity, even when lower concentrations were tested; the same scenario was observed after DAP dosing, with no viable cells found after day 20/25 (Figure S5). The same occurred when dosing WT iPSC-OCs with both drugs, pointing toward drug-specific toxicity. In contrast, amlexanox and ataluren were well tolerated, and no major morphological differences in optic cup structures were found after dosing (Figure 3A).

Full-length PAX6 was detected by western blot in WT and dosed and undosed AN1 iPSC-OC samples on day 35. We observed that 250 μM amlexanox treatment increased full-length PAX6 levels by nearly 4-fold (1.24 ± 0.31, $p < 0.05$) compared with untreated AN1 samples. There was a relative increase in PAX6 in ataluren-treated samples, but it did not reach statistical significance (0.90 ± 0.50, $p = 0.22$) (Figure 3B). Immunostaining confirmed this result, with untreated AN1 showing weaker PAX6 staining in the neural retina layer of untreated AN organoids, which improved after treatment with amlexanox (Figure 3C).

Phenotype rescue in TRID-treated AN iPSC-OCs

To determine whether the increased protein levels following treatment with amlexanox resulted in a functional PAX6 rescue as well as improvement in the molecular and cellular phenotype, we investigated the expression of the key OC marker VSX2. *In vivo*, VSX2 is necessary for establishment of retinal progenitor cells (RPCs) in the OC, and, in the total absence of PAX6, VSX2 expression, along with optic vesicle progression into the OC, is abrogated.[14] Interestingly, AN1 iPSC-OCs showed a 4.08 ± 0.74-fold increase in *VSX2* mRNA levels ($p < 0.001$), and immunostaining confirmed a stronger VSX2 signal in untreated AN1 compared with WT iPSC-OCs (Figures 3D and 3E). After amlexanox and ataluren treatment, *VSX2* expression was significantly downregulated to 1.02 ± 0.67-fold ($p < 0.001$) and 1.05 ± 0.65-fold ($p < 0.001$), respectively, which was indistinguishable from the levels detected in WT samples (WT expression = 1) (Figure 3D). Similarly, immunostaining on day 35 showed weaker VSX2 staining in amlexanox-treated versus untreated AN1 iPSC-OCs. This was less clear for ataluren-treated AN1 iPSC-OCs (Figure 3E).

Cell proliferation alterations have been reported previously in response to abnormal *Pax6* levels.[36,37] Indeed, we observed significant upregulation in *MKI67* expression, which encodes the proliferation marker Ki-67, in AN1 iPSC-OCs compared with the WT (1.65 ± 0.26-fold, $p < 0.01$). This increased proliferative status was also fully rescued after dosing with amlexanox (0.99 ± 0.16, $p < 0.01$) and ataluren (1.07 ± 0.28, $p < 0.05$) (Figure 3F).

TRIDs increase PAX6 protein and improve phenotype in iPSC-LESCs

Because of the reduced PAX6 protein levels already detected in AN iPSC-LESCs on day 15, we dosed cells for 48 h, from day 13 until harvest on day 15.[24] Cells treated with 250 μM amlexanox showed affected viability, so lower concentrations, 100 μM and 200 μM, were used. DAP concentrations of 100 μM and 200 μM did not affect cell viability, and neither did 40 μM ataluren. In contrast, G418 caused significant cell death in iPSC-LESCs, even at doses lower than 100 μg/mL; hence, the readthrough effect could not be analyzed. This was similar to what was observed in the 3D OC models, confirming G418 cytotoxicity.[10,38] Overall, TRID dosing increased full-length PAX6 in AN1 iPSC-LESCs (Figure 4A); amlexanox significantly improved protein levels to 0.650 ± 0.043-fold (100 μM, $p < 0.05$) and 0.941 ± 0.085-fold (200 μM, $p < 0.0001$). Also, 100 μM DAP treatment improved PAX6 levels to 0.912 ± 0.064 ($p < 0.001$). Ataluren-treated cells also showed a significant increase in PAX6, with full-length levels reaching 0.85 ± 0.048-fold ($p < 0.001$) of control levels (Figure 4A). AN2 iPSC-LESCs showed similar trends of increased PAX6 protein when dosed with TRIDs but failed to reach significance when treated with ataluren or amlexanox (Figure 5A). However, 100 μM DAP treatment lead to a significant increase in full-length PAX6 (0.809 ± 0.16-fold, $p < 0.05$) (Figure 5A).

Treatment of AN1 and AN2 iPSC-LESCs with drugs vehicle (DMSO) alone showed no significant changes compared with untreated cells (Figures S6A and S6B). Importantly, dosing of control H9 ESC-derived LESCs with the same TRIDs also showed no effect on PAX6 protein levels, supporting the specificity of these drugs to readthrough of the mutated allele (Figure S6C).

To assess functional and phenotypic rescue following treatment with TRIDs, we examined expression of ABCG2, which is transiently expressed in LESCs and is considered a LESC-specific stemness marker.[39] Although the relationship between PAX6 and ABCG2 is not known, it has been shown recently that *ABCG2* mRNA is upregulated in LESCs extracted from AN patients (with PTC-causing mutations) compared with controls.[40] We observed similar results in our iPSC-derived system, where *ABCG2* mRNA peaked on day 10 in all lines (Figure 2B) and, on day 15, there was a 5.40 ± 1.79-fold accumulation of ABCG2 protein in AN1 and 5.49 ± 1.6-fold in AN2

(C) qRT-PCR transcript analysis of *PAX6* showed no significant difference in expression in AN vs. WT lines. Values were normalized to day 0 and to the internal housekeeping gene *GAPDH*. Data represents means and SD of 3 biological replicates. (D) Protein analysis detected by western blot revealed decreased PAX6 levels between AN1 and AN2 versus WT (WT2 included as an example) samples on day 10 that were statistically significant on day 15 of differentiation. The PAX6/β-actin ratio was normalized to the control (WT). Date expressed at mean ± SD from n = 3 (* $p < 0.05$, t test analysis).

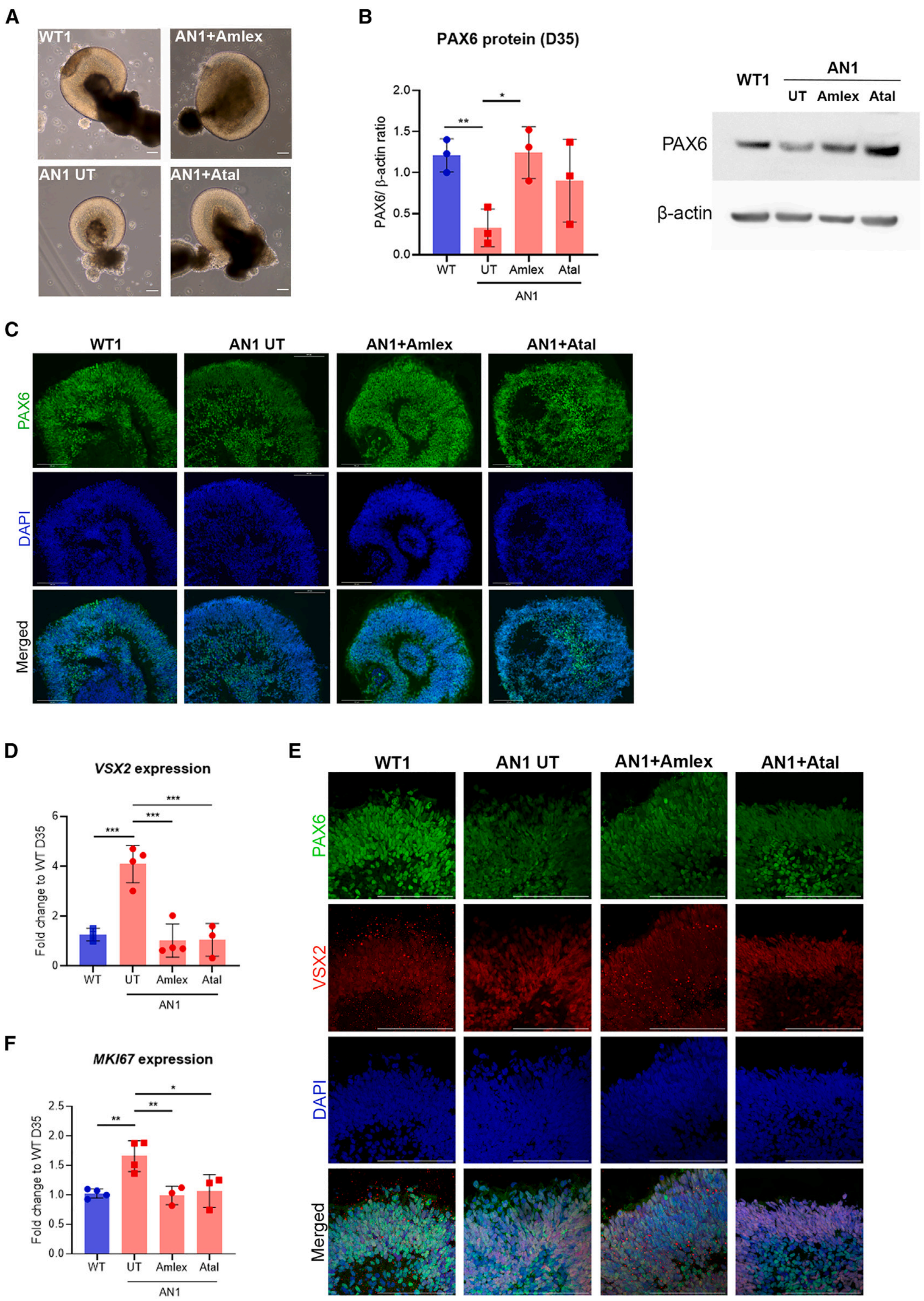

Figure 3. Effect of translational readthrough-inducing drugs (TRIDs) in day 35 AN iPSC-OCs

(A) Bright-field images of control (WT), untreated AN (AN1 UT), amlexanox-treated AN (AN1 Amlex) and ataluren-treated AN (AN1 Atal) iPSC-OCs. Scale bar, 100 μm. (B) Quantification of PAX6 protein in treated vs. UT AN1 iPSC-OCs (red bars). The PAX6/β-actin ratio was normalized to the control (WT, blue bar). $^*p < 0.05$, $^{**}p < 0.01$, one-way

(legend continued on next page)

compared with WT control iPSC-LESCs ($p < 0.05$) (Figures 4B and 5B). Remarkably, amlexanox-treated AN1 iPSC-LESCs showed a very significant reduction in ABCG2 protein, reaching levels very close to the WT, with both concentrations: 100 μM (1.70 ± 0.10-fold, $p < 0.05$) and 200 μM (1.75 ± 0.59, $p < 0.05$) (Figure 4B). Although the same trend was observed for (100 μM) amlexanox-treated AN2 iPSC-LESCs, it did not achieve statistical significance (2.11 ± 1.49-fold, $p = 0.08$) (Figure 5B).

SOX9 and SOX10 are transcription factors expressed in neural crest-fate cells in the limbal niche, which is essential for homeostasis of LESCs.[17] Therefore, and because PAX6 has been shown recently to drive neural crest migration during corneal development,[16,18] we tested the expression of *SOX9* and *SOX10* between AN and WT iPSC-LESCs. Although *SOX9* expression was not significantly altered between AN and the WT (Figures 4C and 5C), we found that *SOX10* was sharply upregulated in both AN iPSC-LESCs (AN1: 20 ± 1.29-fold, $p < 0.001$ [Figure 4D]; AN2: 10.31 ± 1.29 -fold, $p < 0.001$ [Figure 5D]). Following treatment with TRIDs, *SOX10* expression was rescued by 100 μM and 200 μM of amlexanox as well as with ataluren in both patient cell lines (Figures 4D and 5D). Similarly to previous results, DAP did not induce an improvement in *SOX10* expression (Figures 4D and 5D).

In conclusion, amlexanox increases full-length PAX6 levels and rescues phenotypic differences in early 3D OCs and 2D LESCs generated from AN patient iPSCs, proving that newly synthesized PAX6 is functional and that the new amino acid inserted is likely tolerated.

DISCUSSION

The aim of this work is to provide a proof of principle for further development of the repurposed readthrough drugs amlexanox and DAP for aniridia. Aniridia is a highly suitable disease for readthrough therapy approaches because of the high prevalence of *PAX6* nonsense mutations, dosage sensitivity, and, when the target tissue is well considered (i.e., cornea and LESC) to reduce ARK and maintain levels of vision. Insufficient PAX6 levels, or haploinsufficiency, is thought to be the underlying genetic mechanism of aniridia; therefore, increasing full-length PAX6 levels, even if not fully, might be enough to attenuate disease. This is also supported in patients, where PTC-introducing variants are generally associated with severe forms of aniridia, while patients with missense mutations usually present with milder phenotypes and less severe vision loss.[2,3,6]

We generated an iPSC line from an aniridia patient carrying the heterozygous *PAX6* nonsense mutation c.781C>T, p.(Arg261*). This variant is located within the "PAX6 mutation hotspot," a region in exons 8–13 with methylated CpG islands, where 21% of all mutations and 60% of all nonsense mutations are located.[9,41,42]

We differentiated patient iPSCs into 3D OCs (iPSC-OCs) and show significantly reduced PAX6 protein levels on day 35, a time point comparable with the *in vivo* OC stage. Nonetheless, these reduced PAX6 levels are sufficient to form OC domains (neural retina and RPE) in our *in vitro* system, which is also consistent with *in vivo* results.[14] Amlexanox and ataluren have been shown to recover levels of full-length PAX6, while DAP and G418 showed toxicity at all concentrations tested.

We observed a striking increase in neural retina marker VSX2 expression in AN iPSC-derived OCs. Low Pax6 levels seem to promote early neurogenesis in the mouse optic vesicle;[14] this might explain the accumulation of VSX2, which was detected at the mRNA and protein levels. Importantly, we observe that normal VSX2 levels are restored after treatment of AN iPSC-OCs with amlexanox and ataluren. These results suggest that both compounds induce a functional PAX6 protein increase, leading to rescue of the *in vitro* phenotype. This was further supported by downregulation of proliferation marker *MKi67* expression, known to be increased in *Pax6* mutant cells, after dosing of AN iPSC-OCs with both TRIDs.[36]

The role of PAX6 in the eye is time and tissue specific, acting during development but also on maintenance of adult tissue.[14,43] This translates into a developmental and progressive disease, where aniridia patients typically show hypoplasia of the iris and fovea from birth and progressive opacity of the lens and cornea from childhood/early adulthood.[1,9] From large natural history studies, we understand that visual acuity remains relatively stable over decades of life.[3] Therapeutic approaches targeting developmental defects are currently not feasible; hence, we aimed to test the clinical potential of TRIDs to halt or slow down ARK, which can affect up to 90% patients and is the mainstay for a decline in visual acuity over time.[9] For that purpose, we established a second aniridia human model by growing patient iPSC-derived 2D LESCs (iPSC-LESCs). Upregulation of the LESC-specific markers *ΔNP63α*, *ABCG2*, and *KRT14* in these cells proved commitment to the limbal fate. Aniridia patient iPSC-LESCs show over 60% reduction in PAX6 protein levels, lower than the estimated 50%, validating this model to study *PAX6* haploinsufficiency. Again, we did not observe significantly reduced *PAX6* transcript levels in AN vs. WT iPSC-LESCs during the first 15 days of differentiation. It is assumed that *PAX6*-null variants lead to degradation of the mutated transcripts via NMD, resulting in haploinsufficiency;[9] however, we do not seem to observe this in our *in vitro* models; in fact, in the AN iPSC-OCs, we could prove the presence of the mutated

ANOVA. Data represents means and SD of at least 3 biological replicates. (C) Immunofluorescence analysis on day 35 of differentiation, showing PAX6 staining (green) and DAPI (blue) in control (WT), AN1 UT, AN1 Amlex, and AN1 Atal iPSC-OCs. Scale bar, 100 μm. (D) qRT-PCR transcript analysis of the neural retina marker *VSX2* in WT (blue bar) and AN1 UT, AN1 Amlex, and AN1 Atal samples (red bars). *$p < 0.05$, **$p < 0.01$, one-way ANOVA. (E) Immunohistochemical analysis showing VSX2 staining in WT, AN1 UT, and Amlex- and Atal-treated iPSC-OCs. DAPI staining is shown in blue. Scale bar, 100 μm. Red bright spots visible in WT and AN1 Amlex are background staining. (F) qRT-PCR transcript analysis of the proliferation marker *MKi67* in WT (blue bar) and AN1 UT, AN1 Amlex, and AN1 Atal samples (red bars). ***$p < 0.001$, one-way ANOVA. (D and F) Values were normalized to the WT and to the internal housekeeping gene *GAPDH*. Data represents means and SD of at least 3 biological replicates.

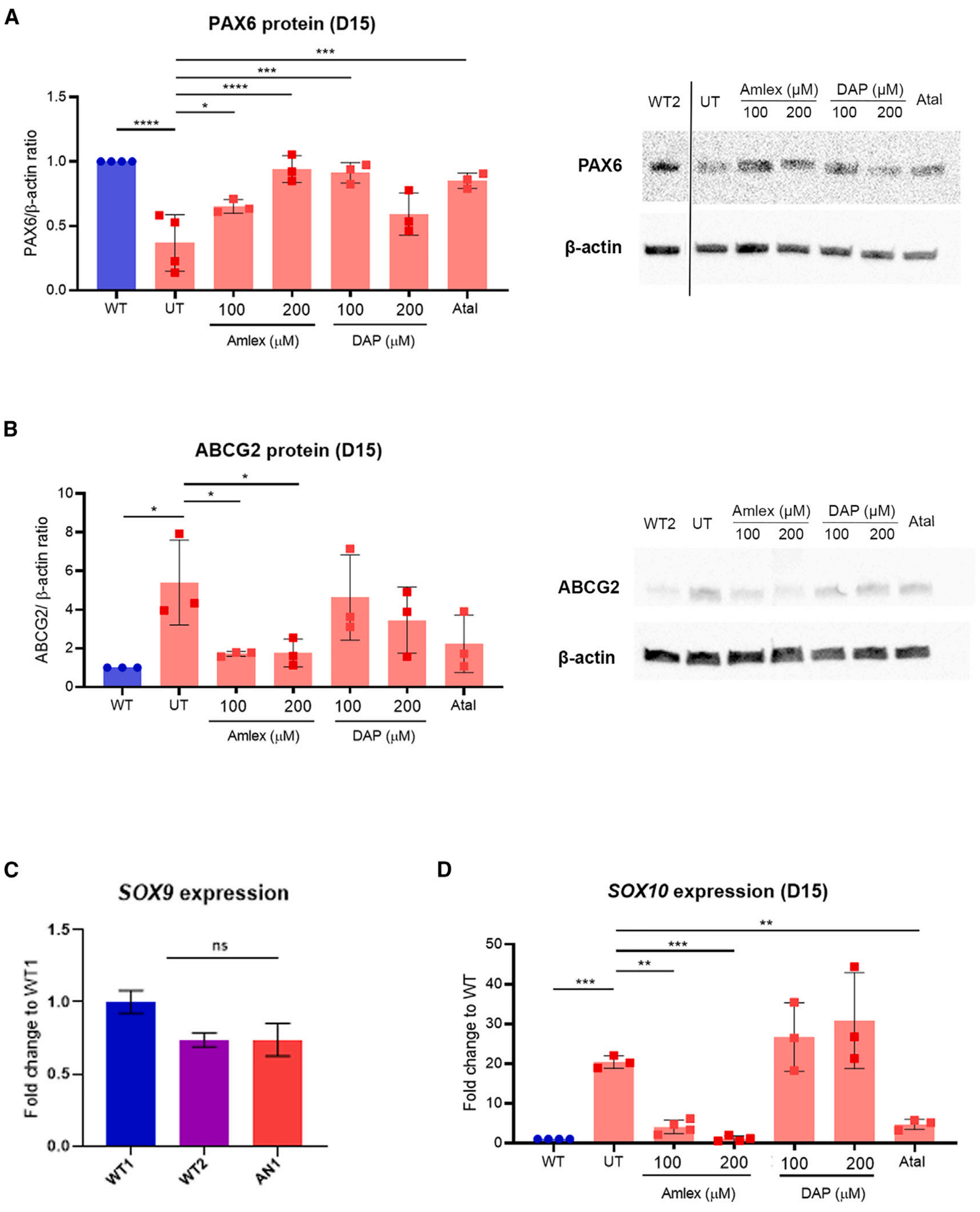

Figure 4. TRIDs rescue PAX6 expression in AN1 iPSC-derived LESCs

(A) Quantification of PAX6 protein in UT AN1 iPSC-LESCs versus Amlex-, DAP-, and Atal-treated AN1 iPSC-LESCs (red bars). The PAX6/β-actin ratio was normalized to the control (WT, blue bar). Values on the x axis refer to compound concentrations (in μM). $^{*}p < 0.05$, $^{***}p < 0.001$, $^{****}p < 0.0001$, one-way ANOVA. Data represents means and SD of at least 3 biological replicates. (B) Quantification of ABCG2 protein detected by western blot in WT (blue bar), AN1 UT, and Amlex-, DAP-, and Atal-treated AN1 iPSC-LESCs (red bars). The PAX6/β-actin ratio was normalized to the control (WT). Data represents means and SD of 3 biological replicates. $^{*}p < 0.05$, one-way ANOVA. (C) Relative expression of *SOX9* transcripts in WT1 and WT2 and AN1 iPSC-LESCs. Significance was calculated using multiple t tests between AN1 and both WT lines. (D) qRT-PCR transcript analysis of the neural crest marker *SOX10* in WT (blue bar) and AN1 UT, AN1 Amlex, and AN1 Atal samples (red bars). $^{***}p < 0.001$, $^{**}p < 0.01$; one-way ANOVA. (C and D) Values were normalized to the WT and to the internal housekeeping gene *GAPDH*. Data represents means and SD of at least 3 biological replicates.

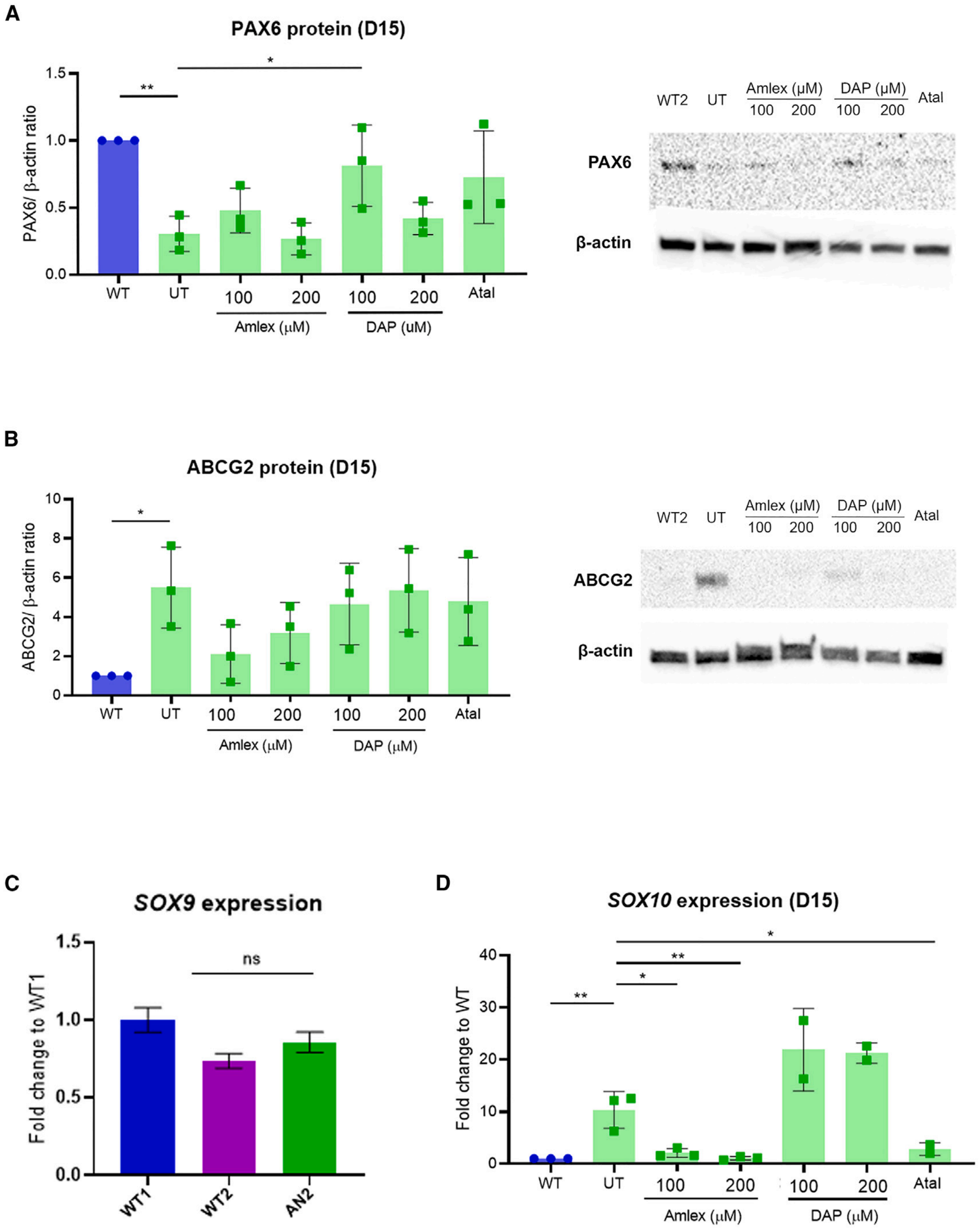

Figure 5. TRIDs show trend improvement in PAX6 expression in AN2 iPSC-derived LESCs

(A) Quantification of PAX6 protein in UT AN2 iPSC-LESCs versus Amlex-, DAP-, and Atal-treated AN2 iPSC-LESCs (green bars). The PAX6/β-actin ratio was normalized to the control (WT, blue bar). Values on the x axis refer to compound concentrations (in μM). *$p < 0.05$, **$p < 0.01$, one-way ANOVA). Data represents means and SD of at least n = 3 biological replicates. (B) Quantification of ABCG2 protein detected by western blot in WT (blue bar), AN2 UT, and Amlex-, DAP-, and Atal-treated AN2 iPSC-LESCs (green bars). Values on the x axis refer to compound concentrations (in μM). The PAX6/β-actin ratio was normalized to the control (WT). Data represent means and SD of 3 biological replicates. *$p < 0.05$, one-way ANOVA. (C) Relative expression of *SOX9* transcripts in WT1, WT2, and AN2 iPSC-LESCs. Significance was calculated using multiple t tests between AN and both WT lines. (D) qRT-PCR transcript analysis of the neural crest marker *SOX10* in WT (blue bar) and AN UT, AN Amlex, and AN Atal samples (green bars). ***$p < 0.001$, **$p < 0.01$, one-way ANOVA. (C and D) Values were normalized to the WT and to the internal housekeeping gene *GAPDH*. Data represents means and SD of at least 3 biological replicates.

transcript, pointing to likely NMD escape. NMD is a multifactorial complex mechanism, and its variable activity has been documented and can vary between patients with the same mutation, as seen in a study involving X-linked choroideremia patients; four individuals with a c.715 C>T; p.(R239*) UGA mutation displayed *CHM* transcript levels ranging from 13%–52.6%.[44] In addition, previous studies have shown that NMD efficiency varies between different murine tissues,[45] but in the choroideremia study, no significant difference in *CHM* mRNA levels were seen between two different patients' fibroblast lines and their corresponding iPSC-derived RPE.[44] The nonsense variants described in this study do result in loss of function, and we therefore hypothesize that other mechanisms could contribute to *PAX6* haploinsufficiency, such as post-translational modifications or epigenetic regulations of the protein;[46,47] this requires further investigation.

Dosing of AN1 iPSC-LESCs with different TRIDs resulted in similar profiles compared with 3D OCs; amlexanox and ataluren proved to significantly increase PAX6 protein, although slightly lower concentrations of amlexanox were used because of very low proliferation in cells treated with 250 μM. Importantly, both compounds, but particularly amlexanox, induced strong phenotype rescue by restoring ABCG2 as well as *SOX10* levels, two important players in LESC identity and survival, respectively.[17,39] Although DAP also induced a significant increase in PAX6 levels in AN1 (and AN2) iPSC-LESCs, it did not show significant downstream phenotypic rescue. We hypothesize this is due to the new amino acid introduced (i.e., tryptophan), which may still have a deleterious effect.[27] Ataluren was well tolerated in both models; in contrast, G418 was highly cytotoxic, proving the downside of traditional aminoglycosides use and need for less toxic TRIDs.[10] DAP showed variable toxicity in iPSC-derived OCs versus LESCs from the same patient (AN1), being cytotoxic in the former and well tolerated and efficient in the latter. Its readthrough ability was reported only very recently, so its mechanism remains unclear.[27] DAP induced a significant increase in PAX6 levels in AN iPSC-LESCs but did not show significant downstream phenotype rescue. We hypothesize that this is due to the new amino acid introduced by the readthrough process; DAP works exclusively with UGA PTC, and tryptophan is the likely substituted amino acid in DAP-mediated readthrough.[27] The likely resultant missense mutations, p.(Arg261Trp) (AN1) and p.(Arg206Trp) (AN2), are predicted to be pathogenic by *in silico* tools; hence, although protein can be detected, it may likely be non/dysfunctional. Reports have shown that missense mutations can lead to milder phenotypes;[3] however, this was not seen at a molecular level and may need *in vivo* studies to confirm this.

ABCG2 is a transient LESC marker, turned off when cells exit the stem cell state and start differentiation into corneal epithelial cells.[39] We hypothesize that increased ABCG2 levels in both AN iPSC-LESCs show that these cells may be unable to switch off their proliferative status and/or trigger the differentiation process into corneal epithelial cells.[40] In parallel, we observe altered expression of the neural crest marker *SOX10*, supporting recent evidence showing that PAX6 has a role in neural crest-derived cells from the limbal niche.[16,18] Further differentiation of AN iPSC-LESCs into later stages as well as high-throughput molecular characterization of these cells would be important to not only understand the mechanisms behind PAX6-related LSCD but also to understand how iPSC-derived models compare with *in vivo* development and disease, particularly when dealing with a regulatory complex transcription factor like PAX6. Importantly, we observed variable efficiency in iPSC differentiation, particularly into LESCs; it is known that there can be substantial inter- and intra-donor iPSC variability,[35] which we tried to address by adding multiple control lines as well as clones for the same line; however, we acknowledge that generation of PAX6 isogenic lines would be an important asset for proving the changes observed in patient cells derived exclusively from PAX6 defects.

Overall, patients with missense *PAX6* mutations tend to have milder ocular phenotypes;[2,6] in our recently published 86-aniridia-patient cohort, patients with missense mutations have a significantly lower incidence of ARK compared with patients with nonsense variants, who present with the highest ARK prevalence.[3] However, the near complete absence of aniridia patients with missense mutations located downstream of exon 7, coupled with the variable expressivity of the disease, makes it difficult to accurately predict the genotype-phenotype relationships. The closest reported missense variant to AN1 c.781C>T, p.(Arg261*) located in exon 10 (predicted homeodomain) was c.773T>C, p.(Phe258Ser); the patient presented with typical iris hypoplasia and chorioretinal coloboma involving the optic disc, but indeed no description of ARK.[48] For the AN2 c.607C>T/p.(Arg203*) variant in exon 8, predicted in the linker region between both DNA-binding domains, the closest reported missense is p.(Arg208Gln), detected in a mid-twenties (at the time of evaluation) female described to have mild symptoms (i.e., nystagmus, foveal hypoplasia, and early cataracts), but again, no ARK was reported.[49]

In this study, we provide strong evidence supporting repurposing of amlexanox as a putative therapeutic compound for aniridia patients with *PAX6* nonsense mutations. Our 3D OC models showed good tolerance to amlexanox, but to reduce off-target effects or systemic complications, topical formulations with a lower dose could be administered.[50] Further work on higher-order *in vivo* models may be required to ascertain the optimal dose needed to induce optimal readthrough in aniridia patients. Interestingly, amlexanox has been shown recently to improve glucose levels and enhance liver fat loss in individuals with type 2 diabetes.[51] Hence, it could be beneficial to assess the effect of systemic amlexanox in aniridia patients because recent reports show that patients commonly present with metabolic dysregulation leading to obesity and type 2 diabetes.[3,52] Therefore, we speculate that amlexanox might have ocular and wider systemic benefits in aniridia patients.

Last, this work provides further evidence that readthrough therapy seems to be a particularly promising therapeutic approach for aniridia, with previous *in vivo* models[12,13] and now patient-specific *in vitro* models showing positive pre-clinical outcomes. The advances in readthrough drug development allied with more complex and

representative human disease models will certainly allow new compounds to be pushed into clinical trials for AN patients.

MATERIALS AND METHODS

Ethics and clinical description

This study was approved by Moorfields Eye Hospital and the National Research Ethics Committee and was conducted in adherence to the tenets of the Declaration of Helsinki; informed written consent was obtained from all participants. 4-mm punch skin biopsies were obtained from the upper arm of a 6-year-old and a 10-year-old male aniridia patient with confirmed genotypes: *PAX6* c.781C>T, p.(Arg261*) (AN1) and c.607C>T/p.(Arg203*) (AN2), respectively. The patient named AN1 was hypermetropic (right eye, +6.00/−2.00 × 10; left eye, +6.00/−1.75 × 180), and the best corrected visual acuity was 0.74 LogMAR in each eye. Intraocular pressure was within the normal range (18 mm Hg in both eyes); there were no signs of glaucoma, cataracts, or ARK, and both corneas were clear. The patient does have complete iris and foveal hypoplasia. Patient AN2 was anisometropic (right eye plano; left eye, −3.50/−3.00 × 160), and his best corrected visual acuity was right eye (RE) 1.6 and left eye (LE) 0.74 LogMAR. Intraocular pressure was RE 28 and LE 27 mm Hg but no signs of glaucoma. He has a history of bilateral cataracts and has had lens extraction, with RE being aphakic and LE receiving an intraocular lens implant. He has right ARK, but the cornea is clear in LE. The patient does have complete iris and foveal hypoplasia.

iPSC generation and culture

Aniridia patient iPSCs were generated using non-integrating episomal reprogramming of dermal fibroblasts extracted from a skin biopsy from the patient's arm, following established protocols.[28,53] A minimum of 2 clonal lines were expanded and characterized as described previously.[29,53] The control (WT) iPSCs used in this study have been published previously.[53] The H9 ESC line was obtained from WiCell (hPSCreg WAe009-A). All iPSC lines were maintained in mTESR Plus medium (STEMCELL Technologies, Canada) with 0.1% penicillin/streptomycin (Pen/Strep) on Matrigel-coated wells (1:100) (Corning, USA). For passaging, ReLESR (STEMCELL Technologies) was used for detaching, and after 24 h, iPSCs were fed daily with mTESR Plus until confluent.

iPSC differentiation into 3D OCs

Differentiation of iPSCs into 3D OCs was performed based on published protocols.[30,31] Briefly, confluent iPSCs were detached with Accumax (Thermo Fisher Scientific, MA, USA) to single-cell suspension, and 3.6 million cells per well were plated onto Aggrewell400 plates (STEMCELL Technologies) (3,000 cells per microwell) in mTESR Plus with 10 μM Y-27632 (Abcam), following the manufacturer's instructions. After 48 h, EBs were collected and plated onto low-attachment 60-mm^2 plates in neural induction medium (NIM; DMEM/F12 [Thermo Fisher Scientific], 20% knockout serum replacement [KOSR; Thermo Fisher Scientific], 2% B27 [Thermo Fisher Scientific], 1× non-essential amino acids [NEAAs; Thermo Fisher Scientific], 1% Pen/Strep, 1× GlutaMAX [Thermo Fisher Scientific], and 5 ng/mL IGF-1 [Sigma-Aldrich, USA]) until day 7. On day 8, cells were cultured in NIM with 15% KOSR and finally with 10% KOSR from day 11 until day 35 (Figure 1B).

iPSC differentiation into LESCs

Differentiation of iPSCs into LESCs was done following the protocol from Hongisto et al.[34] with small adjustments. Confluent iPSCs (∼90%/95%) were detached using ReLESR, and clumps were resuspended in mTESR Plus with 10 μM Y-27632. Cell clumps were transferred into non-coated (Petri) dishes and incubated overnight to allow formation of EBs (day 0). After 48 h (day 2), EBs were carefully washed with Dulbecco's phosphate buffered saline (DPBS) and resuspended in SM medium (KnockOut DMEM supplemented with 15% xeno-free serum replacement, 2 mM L-glutamine, 0.1 mM 2-mercaptoethanol, 1% NEAAs, and 50 U/mL Pen/Strep) supplemented with 10 μM SB-505124 (Sigma-Aldrich) and 50 ng/mL bFGF (Peprotech, USA). Medium was replaced with SM medium supplemented with 25 ng/mL BMP-4 (Peprotech) on days 3 and 4. On day 5, EBs were carefully plated into collagen IV-coated wells in a mix of CnT30 medium (CellnTech, Switzerland) and SM medium (3:1) and allowed to attach for 48 h. From there on, medium was changed with CnT30 every other day until collection on day 15 for RNA and protein analysis.

Dosing and compound information

The dosing concentrations of amlexanox (Abcam) and DAP (Sigma-Aldrich) were based on previous publications.[24,26,27] The known TRIDs ataluren/PTC124 (ApexBio Tech) and G418 (Life Technologies) were used as positive readthrough controls at 40 μM and 100 μg/mL, respectively, according to previous publications from our group.[25,44,54] 3D OCs were dosed with amlexanox (250 μM) or ataluren (40 μM) in NIM + 10% KOSR from days 15–35 of differentiation, with medium refreshed every other day. iPSC-LESCs were dosed from days 13–15 of differentiation in CnT-30 medium, with medium change after 24 h. Amlexanox (100 μM and 200 μM), DAP (100 μM and 200 μM), ataluren (40 μM), and G418 (100 μg/mL) were tested.

RNA extraction and qRT-PCR

For transcript analysis of 3D OCs, RNA extraction was performed after pellet collection using the RNeasy Mini or Micro Kit (QIAGEN, Germany); iPSC-LESCs were harvested by adding 300 μL of lysis buffer (Zymo Research, USA), and cells were collected using a cell scraper. RNA was extracted following instructions in the Quick-RNA MicroPrep Kit w/Zymo-Spin IC Columns Kit (Zymo Research).

cDNA was synthesized from 500 ng RNA using the High-Capacity RNA-to-cDNA Kit (Life Technologies). qRT-PCR was performed with 2× SYBR Green MasterMix (Thermo Fisher Scientific) on a StepOne real-time PCR system (Applied Biosystems, UK) or QuantStudio 6 Flex (Applied Biosystems). Primers used for qPCR are listed in Table S1. Transcript levels were measured in duplicate and normalized to the housekeeping gene *GAPDH* or *ACTB*. The relative expression of each target gene was calculated using the comparative C_T method.

Western blotting

Samples were analyzed by western blotting as described previously.[25,55] Cells were washed with ice-cold PBS, and total protein extract was prepared with RIPA buffer with 1× Halt protease inhibitor cocktail and Halt phosphatase inhibitor (Thermo Fisher Scientific) at a ratio of 5 × 106 cells/mL. 30 μg protein for iPSC-derived OCs or 15 μg for iPSC-LESCs was loaded onto 4%–15% Mini-PROTEAN TGX gels (Bio-Rad, CA, USA) and transferred to an Immun-Blot polyvinylidene fluoride (PVDF) membrane using a Trans-Blot SD semi-dry transfer cell (Bio-Rad). Membranes were blocked with 5% non-fat dry milk in PBS-Tween 20 (0.1%) for 2 h and incubated overnight at 4°C with the following primary antibodies diluted in blocking buffer: PAX6 (1:2,000, Covance), ABCG2 (1:1,000, Santa Cruz Biotechnology), and β-actin (1:5,000, Sigma-Aldrich). Incubation with horseradish peroxidase-conjugated secondary antibody (anti-mouse or -rabbit, 1:5,000, Applied Biosystems) was done for 2 h at room temperature. Membranes were incubated with Clarity Western ECL Substrate (Bio-Rad) and imaged using the ChemiDoc XRS Imaging System (Bio-Rad). Band intensities were quantified using the Fiji/ImageJ software (National Institutes of Health, MD, USA).

Immunofluorescence and imaging

Day 35 iPSC-OCs were processed for immunohistochemistry analysis following the protocol from Reichman and Goureau.[56] Slides were imaged using an EVOS FL system (Thermo Fisher Scientific) and LSM 700 or LSM 710 (Carl Zeiss, Germany).

Statistical analysis

Statistical analysis was performed using Prism 8.0 (GraphPad, San Diego, CA, USA). One-Way ANOVA with multiple comparisons was used for comparison studies, with significance achieved as follows: *$p \leq 0.05$, **$p \leq 0.01$, ***$p \leq 0.001$. All results are expressed as mean ± SD unless specified otherwise. Experiments were performed with 3 biological replicates, except when specified otherwise.

DATA AND CODE AVAILABILITY

Data sharing is not applicable to this article because no datasets were generated or analyzed during the current study.

SUPPLEMENTAL INFORMATION

Supplemental information can be found online at https://doi.org/10.1016/j.omtn.2023.06.016.

ACKNOWLEDGMENTS

The authors would like to thank the patients and their families for donating skin samples. Funding for this work was obtained from the Moorfields Eye Charity, the Wellcome Trust (205174/Z/16/Z), Fight for Sight, and the Aniridia Network (to M.M.) and EJP-RD (JPRD20-135) and an NWO visitor travel grant (040.11.699) (to J.H.Z. and D.L.C.).

AUTHOR CONTRIBUTIONS

M.M. designed research and acquired funding. D.L.C., H.S., J.E., and P.H. performed research. J.H.Z. contributed reagents and to research design. D.L.C. and M.M. analyzed data. D.L.C. wrote the first draft. D.L.C. and M.M. edited the manuscript with input from all authors.

DECLARATION OF INTERESTS

The authors declare no competing interests.

REFERENCES

1. Moosajee, M., Hingorani, M., and Moore, A.T. (2018). PAX6-Related Aniridia. In GeneReviews(®) (University of Washington, Seattle).
2. Hingorani, M., Williamson, K.A., Moore, A.T., and van Heyningen, V. (2009). Detailed ophthalmologic evaluation of 43 individuals with PAX6 mutations. Invest. Ophthalmol. Vis. Sci. *50*, 2581–2590. https://doi.org/10.1167/iovs.08-2827.
3. Kit, V., Lima Cunha, D., Hagag, A.M., and Moosajee, M. (2021). Longitudinal genotype-phenotype analysis in 86 PAX6-related aniridia patients. JCI insight. https://doi.org/10.1172/jci.insight.148406.
4. Daruich, A., Duncan, M., Robert, M.P., Lagali, N., Semina, E.V., Aberdam, D., Ferrari, S., Romano, V., des Roziers, C.B., Benkortebi, R., et al. (2022). Congenital aniridia beyond black eyes: From phenotype and novel genetic mechanisms to innovative therapeutic approaches. Prog. Retin. Eye Res.101133. https://doi.org/10.1016/j.preteyeres.2022.101133.
5. Latta, L., Figueiredo, F.C., Ashery-Padan, R., Collinson, J.M., Daniels, J., Ferrari, S., Szentmáry, N., Solá, S., Shalom-Feuerstein, R., Lako, M., et al. (2021). Pathophysiology of aniridia-associated keratopathy: Developmental aspects and unanswered questions. Ocul. Surf. *22*, 245–266. https://doi.org/10.1016/j.jtos.2021.09.001.
6. Lagali, N., Wowra, B., Fries, F.N., Latta, L., Moslemani, K., Utheim, T.P., Wylegala, E., Seitz, B., and Käsmann-Kellner, B. (2020). PAX6 Mutational Status Determines Aniridia-Associated Keratopathy Phenotype. Ophthalmology *127*, 273–275. https://doi.org/10.1016/j.ophtha.2019.09.034.
7. Jordan, T., Hanson, I., Zaletayev, D., Hodgson, S., Prosser, J., Seawright, A., Hastie, N., and van Heyningen, V. (1992). The human PAX6 gene is mutated in two patients with aniridia. Nat. Genet. *1*, 328–332. https://doi.org/10.1038/ng0892-328.
8. Hill, R.E., Favor, J., Hogan, B.L., Ton, C.C., Saunders, G.F., Hanson, I.M., Prosser, J., Jordan, T., Hastie, N.D., and van Heyningen, V. (1991). Mouse small eye results from mutations in a paired-like homeobox-containing gene. Nature *354*, 522–525. https://doi.org/10.1038/354522a0.
9. Lima Cunha, D., Arno, G., Corton, M., and Moosajee, M. (2019). The Spectrum of PAX6 Mutations and Genotype-Phenotype Correlations in the Eye. Genes *10*, 1050. https://doi.org/10.3390/genes10121050.
10. Way, C.M., Lima Cunha, D., and Moosajee, M. (2020). Translational readthrough inducing drugs for the treatment of inherited retinal dystrophies. Expet Rev. Ophthalmol. *15*, 169–182. https://doi.org/10.1080/17469899.2020.1762489.
11. Richardson, R., Smart, M., Tracey-White, D., Webster, A.R., and Moosajee, M. (2017). Mechanism and evidence of nonsense suppression therapy for genetic eye disorders. Exp. Eye Res. *155*, 24–37. https://doi.org/10.1016/j.exer.2017.01.001.
12. Gregory-Evans, C.Y., Wang, X., Wasan, K.M., Zhao, J., Metcalfe, A.L., and Gregory-Evans, K. (2014). Postnatal manipulation of Pax6 dosage reverses congenital tissue malformation defects. J. Clin. Invest. *124*, 111–116. https://doi.org/10.1172/jci70462.
13. Wang, X., Gregory-Evans, K., Wasan, K.M., Sivak, O., Shan, X., and Gregory-Evans, C.Y. (2017). Efficacy of Postnatal In Vivo Nonsense Suppression Therapy in a Pax6 Mouse Model of Aniridia. Molecular therapy. Nucleic acids *7*, 417–428. https://doi.org/10.1016/j.omtn.2017.05.002.
14. Shaham, O., Menuchin, Y., Farhy, C., and Ashery-Padan, R. (2012). Pax6: a multi-level regulator of ocular development. Prog. Retin. Eye Res. *31*, 351–376. https://doi.org/10.1016/j.preteyeres.2012.04.002.
15. Collinson, J.M., Chanas, S.A., Hill, R.E., and West, J.D. (2004). Corneal development, limbal stem cell function, and corneal epithelial cell migration in the Pax6(+/-) mouse. Invest. Ophthalmol. Vis. Sci. *45*, 1101–1108. https://doi.org/10.1167/iovs.03-1118.
16. Chen, S.Y., Cheng, A.M.S., Zhang, Y., Zhu, Y.T., He, H., Mahabole, M., and Tseng, S.C.G. (2019). Pax 6 Controls Neural Crest Potential of Limbal Niche Cells to

Support Self-Renewal of Limbal Epithelial Stem Cells. Sci. Rep. *9*, 9763. https://doi.org/10.1038/s41598-019-45100-7.

17. Su, Z., Wang, J., Lai, Q., Zhao, H., and Hou, L. (2020). KIT ligand produced by limbal niche cells under control of SOX10 maintains limbal epithelial stem cell survival by activating the KIT/AKT signalling pathway. J. Cell Mol. Med. *24*, 12020–12031. https://doi.org/10.1111/jcmm.15830.

18. Takamiya, M., Stegmaier, J., Kobitski, A.Y., Schott, B., Weger, B.D., Margariti, D., Cereceda Delgado, A.R., Gourain, V., Scherr, T., Yang, L., et al. (2020). Pax6 organizes the anterior eye segment by guiding two distinct neural crest waves. PLoS Genet. *16*, e1008774. https://doi.org/10.1371/journal.pgen.1008774.

19. Takahashi, K., Tanabe, K., Ohnuki, M., Narita, M., Ichisaka, T., Tomoda, K., and Yamanaka, S. (2007). Induction of pluripotent stem cells from adult human fibroblasts by defined factors. Cell *131*, 861–872. https://doi.org/10.1016/j.cell.2007.11.019.

20. Hata, M., Ikeda, H.O., Iwai, S., Iida, Y., Gotoh, N., Asaka, I., Ikeda, K., Isobe, Y., Hori, A., Nakagawa, S., et al. (2018). Reduction of lipid accumulation rescues Bietti's crystalline dystrophy phenotypes. Proc. Natl. Acad. Sci. USA *115*, 3936–3941. https://doi.org/10.1073/pnas.1717338115.

21. Lane, A., Jovanovic, K., Shortall, C., Ottaviani, D., Panes, A.B., Schwarz, N., Guarascio, R., Hayes, M.J., Palfi, A., Chadderton, N., et al. (2020). Modeling and Rescue of RP2 Retinitis Pigmentosa Using iPSC-Derived Retinal Organoids. Stem Cell Rep. *15*, 67–79. https://doi.org/10.1016/j.stemcr.2020.05.007.

22. Ramsden, C.M., Nommiste, B., R Lane, A., Carr, A.J.F., Powner, M.B., J K Smart, M., Chen, L.L., Muthiah, M.N., Webster, A.R., Moore, A.T., et al. (2017). Rescue of the MERTK phagocytic defect in a human iPSC disease model using translational read-through inducing drugs. Sci. Rep. *7*, 51. https://doi.org/10.1038/s41598-017-00142-7.

23. Greer, R.O., Jr., Lindenmuth, J.E., Juarez, T., and Khandwala, A. (1993). A double-blind study of topically applied 5% amlexanox in the treatment of aphthous ulcers. J. Oral Maxillofac. Surg. *51*, 243–248. https://doi.org/10.1016/s0278-2391(10)80164-8.

24. Atanasova, V.S., Jiang, Q., Prisco, M., Gruber, C., Piñón Hofbauer, J., Chen, M., Has, C., Bruckner-Tuderman, L., McGrath, J.A., Uitto, J., and South, A.P. (2017). Amlexanox Enhances Premature Termination Codon Read-Through in COL7A1 and Expression of Full Length Type VII Collagen: Potential Therapy for Recessive Dystrophic Epidermolysis Bullosa. J. Invest. Dermatol. *137*, 1842–1849. https://doi.org/10.1016/j.jid.2017.05.011.

25. Eintracht, J., Forsythe, E., May-Simera, H., and Moosajee, M. (2021). Translational readthrough of ciliopathy genes BBS2 and ALMS1 restores protein, ciliogenesis and function in patient fibroblasts. EBioMedicine *70*, 103515. https://doi.org/10.1016/j.ebiom.2021.103515.

26. Gonzalez-Hilarion, S., Beghyn, T., Jia, J., Debreuck, N., Berte, G., Mamchaoui, K., Mouly, V., Gruenert, D.C., Déprez, B., and Lejeune, F. (2012). Rescue of nonsense mutations by amlexanox in human cells. Orphanet J. Rare Dis. *7*, 58. https://doi.org/10.1186/1750-1172-7-58.

27. Trzaska, C., Amand, S., Bailly, C., Leroy, C., Marchand, V., Duvernois-Berthet, E., Saliou, J.M., Benhabiles, H., Werkmeister, E., Chassat, T., et al. (2020). 2,6-Diaminopurine as a highly potent corrector of UGA nonsense mutations. Nat. Commun. *11*, 1509. https://doi.org/10.1038/s41467-020-15140-z.

28. Parfitt, D.A., Lane, A., Ramsden, C.M., Carr, A.J.F., Munro, P.M., Jovanovic, K., Schwarz, N., Kanuga, N., Muthiah, M.N., Hull, S., et al. (2016). Identification and Correction of Mechanisms Underlying Inherited Blindness in Human iPSC-Derived Optic Cups. Cell Stem Cell *18*, 769–781. https://doi.org/10.1016/j.stem.2016.03.021.

29. Harding, P., Lima Cunha, D., Méjécase, C., Eintracht, J., Toualbi, L., Sarkar, H., and Moosajee, M. (2021). Generation of human iPSC line (UCLi013-A) from a patient with microphthalmia and aniridia, carrying a heterozygous missense mutation c.372C>A p.(Asn124Lys) in PAX6. Stem Cell Res. *51*, 102184. https://doi.org/10.1016/j.scr.2021.102184.

30. Mellough, C.B., Collin, J., Khazim, M., White, K., Sernagor, E., Steel, D.H.W., and Lako, M. (2015). IGF-1 Signaling Plays an Important Role in the Formation of Three-Dimensional Laminated Neural Retina and Other Ocular Structures From Human Embryonic Stem Cells. Stem Cell. *33*, 2416–2430. https://doi.org/10.1002/stem.2023.

31. Eintracht, J., Harding, P., Lima Cunha, D., and Moosajee, M. (2022). Efficient embryoid-based method to improve generation of optic vesicles from human induced pluripotent stem cells. F1000Res. *11*, 324. https://doi.org/10.12688/f1000research.108829.1.

32. Nakano, T., Ando, S., Takata, N., Kawada, M., Muguruma, K., Sekiguchi, K., Saito, K., Yonemura, S., Eiraku, M., and Sasai, Y. (2012). Self-formation of optic cups and storable stratified neural retina from human ESCs. Cell Stem Cell *10*, 771–785. https://doi.org/10.1016/j.stem.2012.05.009.

33. Zhong, X., Gutierrez, C., Xue, T., Hampton, C., Vergara, M.N., Cao, L.H., Peters, A., Park, T.S., Zambidis, E.T., Meyer, J.S., et al. (2014). Generation of three-dimensional retinal tissue with functional photoreceptors from human iPSCs. Nat. Commun. *5*, 4047. https://doi.org/10.1038/ncomms5047.

34. Hongisto, H., Vattulainen, M., Ilmarinen, T., Mikhailova, A., and Skottman, H. (2018). Efficient and Scalable Directed Differentiation of Clinically Compatible Corneal Limbal Epithelial Stem Cells from Human Pluripotent Stem Cells. J. Vis. Exp.58279. https://doi.org/10.3791/58279.

35. Beekhuis-Hoekstra, S.D., Watanabe, K., Werme, J., de Leeuw, C.A., Paliukhovich, I., Li, K.W., Koopmans, F., Smit, A.B., Posthuma, D., and Heine, V.M. (2021). Systematic assessment of variability in the proteome of iPSC derivatives. Stem Cell Res. *56*, 102512. https://doi.org/10.1016/j.scr.2021.102512.

36. Rabiee, B., Anwar, K.N., Shen, X., Putra, I., Liu, M., Jung, R., Afsharkhamseh, N., Rosenblatt, M.I., Fishman, G.A., Liu, X., et al. (2020). Gene dosage manipulation alleviates manifestations of hereditary PAX6 haploinsufficiency in mice. Sci. Transl. Med. *12*, eaaz4894. https://doi.org/10.1126/scitranslmed.aaz4894.

37. Ouyang, J., Shen, Y.C., Yeh, L.K., Li, W., Coyle, B.M., Liu, C.Y., and Fini, M.E. (2006). Pax6 overexpression suppresses cell proliferation and retards the cell cycle in corneal epithelial cells. Invest. Ophthalmol. Vis. Sci. *47*, 2397–2407. https://doi.org/10.1167/iovs.05-1083.

38. Hancock, H.A., Guidry, C., Read, R.W., Ready, E.L., and Kraft, T.W. (2005). Acute aminoglycoside retinal toxicity in vivo and in vitro. Invest. Ophthalmol. Vis. Sci. *46*, 4804–4808. https://doi.org/10.1167/iovs.05-0604.

39. Vattulainen, M., Ilmarinen, T., Koivusalo, L., Viiri, K., Hongisto, H., and Skottman, H. (2019). Modulation of Wnt/BMP pathways during corneal differentiation of hPSC maintains ABCG2-positive LSC population that demonstrates increased regenerative potential. Stem Cell Res. Ther. *10*, 236. https://doi.org/10.1186/s13287-019-1354-2.

40. Latta, L., Nordström, K., Stachon, T., Langenbucher, A., Fries, F.N., Szentmáry, N., Seitz, B., and Käsmann-Kellner, B. (2019). Expression of retinoic acid signaling components ADH7 and ALDH1A1 is reduced in aniridia limbal epithelial cells and a siRNA primary cell based aniridia model. Exp. Eye Res. *179*, 8–17. https://doi.org/10.1016/j.exer.2018.10.002.

41. Tzoulaki, I., White, I.M.S., and Hanson, I.M. (2005). PAX6 mutations: genotype-phenotype correlations. BMC Genet. *6*, 27. https://doi.org/10.1186/1471-2156-6-27.

42. Wawrocka, A., and Krawczynski, M.R. (2018). The genetics of aniridia - simple things become complicated. J. Appl. Genet. *59*, 151–159. https://doi.org/10.1007/s13353-017-0426-1.

43. Marquardt, T., Ashery-Padan, R., Andrejewski, N., Scardigli, R., Guillemot, F., and Gruss, P. (2001). Pax6 is required for the multipotent state of retinal progenitor cells. Cell *105*, 43–55. https://doi.org/10.1016/s0092-8674(01)00295-1.

44. Sarkar, H., Mitsios, A., Smart, M., Skinner, J., Welch, A.A., Kalatzis, V., Coffey, P.J., Dubis, A.M., Webster, A.R., and Moosajee, M. (2019). Nonsense-mediated mRNA decay efficiency varies in choroideremia providing a target to boost small molecule therapeutics. Hum. Mol. Genet. *28*, 1865–1871. https://doi.org/10.1093/hmg/ddz028.

45. Zetoune, A.B., Fontanière, S., Magnin, D., Anczuków, O., Buisson, M., Zhang, C.X., and Mazoyer, S. (2008). Comparison of nonsense-mediated mRNA decay efficiency in various murine tissues. BMC Genet. *9*, 83. https://doi.org/10.1186/1471-2156-9-83.

46. Yu, F., Zhang, W., Yan, C., Yan, D., Zhou, M., Chen, J., Zhao, X., Zhu, A., Zhou, J., Liu, H., et al. (2020). PAX6, modified by SUMOylation, plays a protective role in corneal endothelial injury. Cell Death Dis. *11*, 683. https://doi.org/10.1038/s41419-020-02848-5.

47. Tsui, S., Wang, J., Wang, L., Dai, W., and Lu, L. (2016). CTCF-Mediated and Pax6-Associated Gene Expression in Corneal Epithelial Cell-Specific Differentiation. PLoS One *11*, e0162071. https://doi.org/10.1371/journal.pone.0162071.

48. Azuma, N., Yamaguchi, Y., Handa, H., Tadokoro, K., Asaka, A., Kawase, E., and Yamada, M. (2003). Mutations of the PAX6 gene detected in patients with a variety of optic-nerve malformations. Am. J. Hum. Genet. *72*, 1565–1570. https://doi.org/10.1086/375555.

49. Grønskov, K., Rosenberg, T., Sand, A., and Brøndum-Nielsen, K. (1999). Mutational analysis of PAX6: 16 novel mutations including 5 missense mutations with a mild aniridia phenotype. Eur. J. Hum. Genet. *7*, 274–286. https://doi.org/10.1038/sj.ejhg.5200308.

50. Woodley, D.T., Cogan, J., Hou, Y., Lyu, C., Marinkovich, M.P., Keene, D., and Chen, M. (2017). Gentamicin induces functional type VII collagen in recessive dystrophic epidermolysis bullosa patients. J. Clin. Invest. *127*, 3028–3038. https://doi.org/10.1172/jci92707.

51. Oral, E.A., Reilly, S.M., Gomez, A.V., Meral, R., Butz, L., Ajluni, N., Chenevert, T.L., Korytnaya, E., Neidert, A.H., Hench, R., et al. (2017). Inhibition of IKKϵ and TBK1 Improves Glucose Control in a Subset of Patients with Type 2 Diabetes. Cell Metabol. *26*, 157–170.e7. https://doi.org/10.1016/j.cmet.2017.06.006.

52. Netland, P.A., Scott, M.L., Boyle, J.W., 4th, and Lauderdale, J.D. (2011). Ocular and systemic findings in a survey of aniridia subjects. J. AAPOS *15*, 562–566. https://doi.org/10.1016/j.jaapos.2011.07.009.

53. Méjécase, C., Harding, P., Sarkar, H., Eintracht, J., Lima Cunha, D., Toualbi, L., and Moosajee, M. (2020). Generation of two human control iPS cell lines (UCLi016-A and UCLi017-A) from healthy donors with no known ocular conditions. Stem Cell Res. *49*, 102113. https://doi.org/10.1016/j.scr.2020.102113.

54. Torriano, S., Erkilic, N., Baux, D., Cereso, N., De Luca, V., Meunier, I., Moosajee, M., Roux, A.F., Hamel, C.P., and Kalatzis, V. (2018). The effect of PTC124 on choroideremia fibroblasts and iPSC-derived RPE raises considerations for therapy. Sci. Rep. *8*, 8234. https://doi.org/10.1038/s41598-018-26481-7.

55. Moosajee, M., Tracey-White, D., Smart, M., Weetall, M., Torriano, S., Kalatzis, V., da Cruz, L., Coffey, P., Webster, A.R., and Welch, E. (2016). Functional rescue of REP1 following treatment with PTC124 and novel derivative PTC-414 in human choroideremia fibroblasts and the nonsense-mediated zebrafish model. Hum. Mol. Genet. *25*, 3416–3431. https://doi.org/10.1093/hmg/ddw184.

56. Reichman, S., and Goureau, O. (2016). Production of Retinal Cells from Confluent Human iPS Cells. Methods Mol. Biol. *1357*, 339–351. https://doi.org/10.1007/7651_2014_143.

OMTN, Volume *33*

Supplemental information

Restoration of functional PAX6 in aniridia patient iPSC-derived ocular tissue models using repurposed nonsense suppression drugs

Dulce Lima Cunha, Hajrah Sarkar, Jonathan Eintracht, Philippa Harding, Jo Huiqing Zhou, and Mariya Moosajee

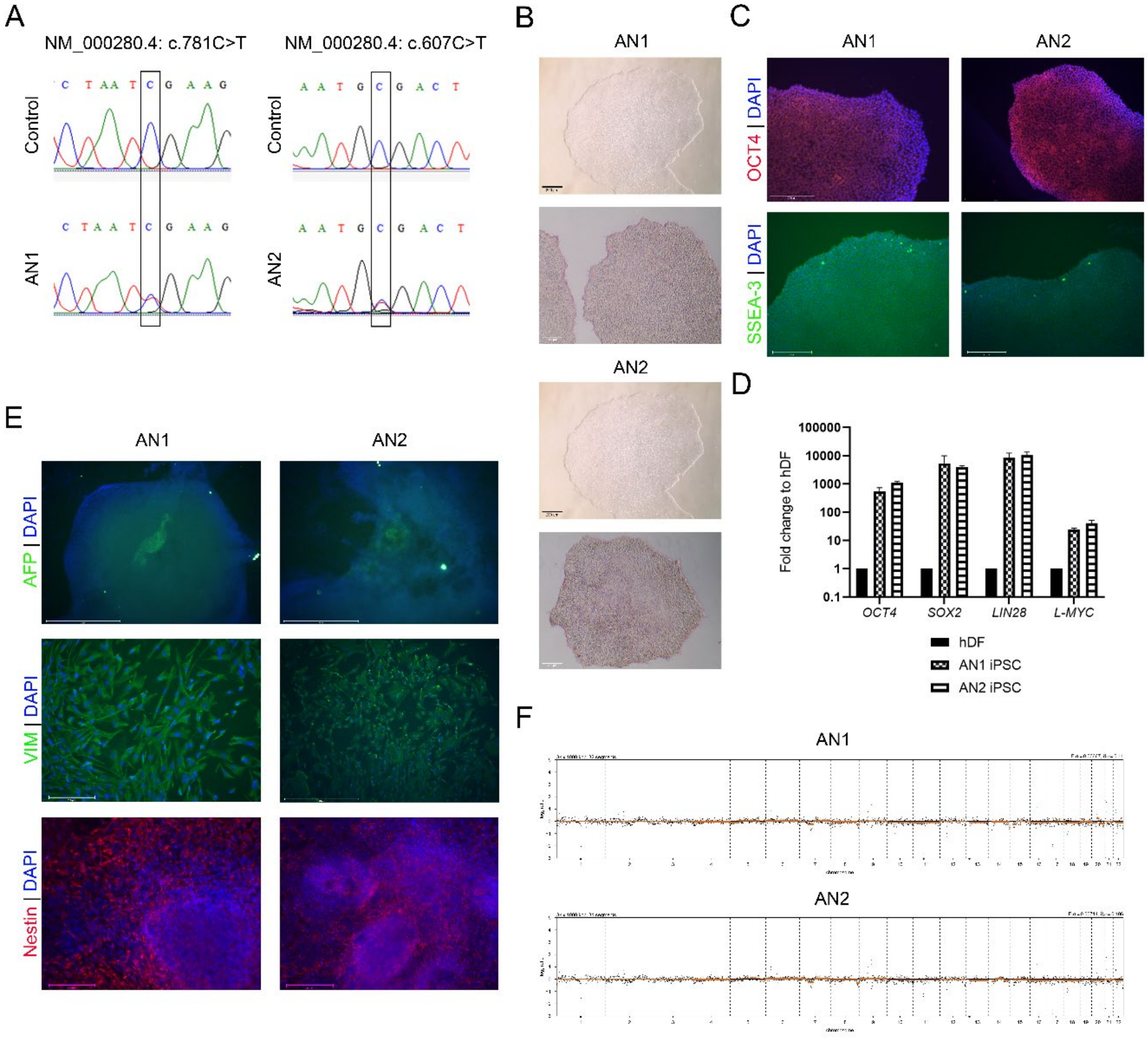

Figure S1. Characterisation of iPSCs generated from two independent aniridia patient (AN1 and AN2) carrying *PAX6* heterozygous nonsense variants. (A) The heterozygous nonsense variants c.781C>T, p.Arg261* (AN1) and c.607C>T, p.(Arg203*) (AN2) in the *PAX6* gene (NM_000280.4) were confirmed in each aniridia iPSC line through direct sequencing and were absent from control iPSCs. (B) Embryonic stem cell-like morphology and positive alkaline phosphatase (red) staining. (C) Positive expression of pluripotency markers OCT4 (upper panel) and SSEA-3 (lower panel). Scale bar 200µm. (D) Pluripotency marker genes *OCT4*, *SOX2*, *L-MYC* and *LIN28* were upregulated in both AN iPSC compared to each parental fibroblasts (hDF) line by qRT-PCR. (E) In vitro differentiation ability was confirmed by random differentiation of both aniridia iPSCs: cells

stained positive for endoderm (AFP), mesoderm (Vimentin) and ectoderm (Nestin) markers. (F) Low-pass whole genome sequencing analysis revealed no abnormalities in AN1 and AN2 iPSCs, showing 46,XY karyotype in both cases.

A

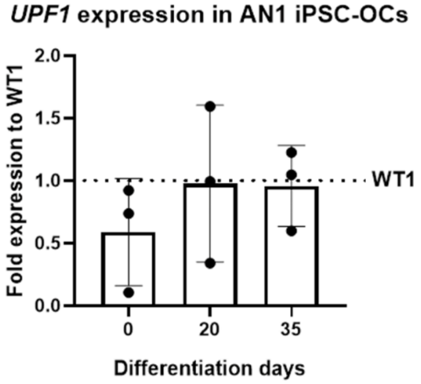

B

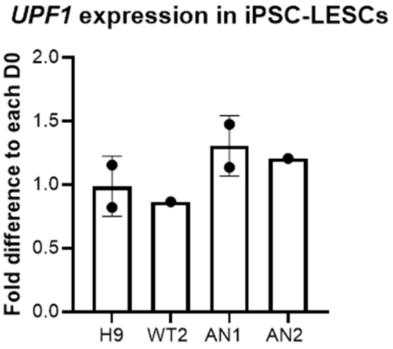

Figure S2. NMD status in iPSC-derived models. (A) *UPF1* mRNA expression levels in AN1 iPSC-OCs were measured by qRT-PCR on differentiation days 0, 20 and 35 and normalised to WT1 expression on the same day (dotted line). No significant changes were detected (n=3). (B) *UPF1* mRNA expression in control (H9 and WT2) and aniridia (AN1 and AN2) iPSC-LESCs on differentiation day 15 and normalised to each line's day 0.

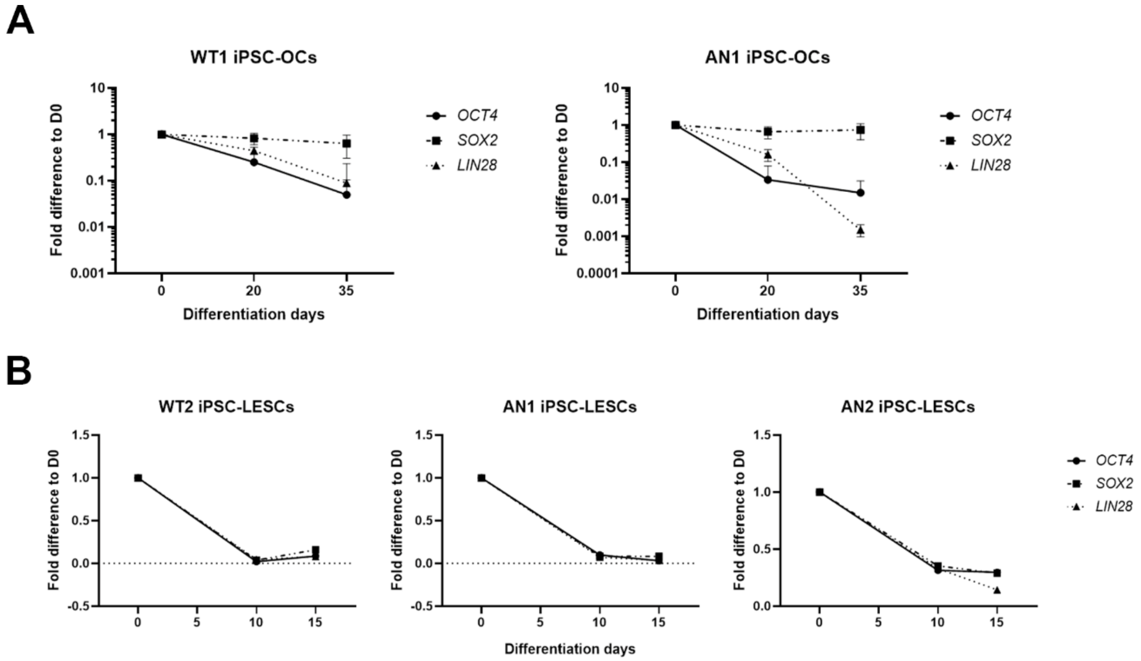

Figure S3. Pluripotency genes expression during WT and AN differentiation. (A) Expression of *OCT4*, *SOX2* and *LIN28* was detected by qRT-PCR on days 0, 20 and 35 of optic cup (OCs) differentiation. *OCT4* and *LIN28* were significantly downregulated; SOX2 is required for early eye development, hence no mRNA reduction is seen compared to day 0. (B) All three markers were downregulated during iPSC differentiation into limbal epithelial stem cells (LESCs), for all lines tested. Values were normalised to day 0 and to internal housekeeper gene *GAPDH.*

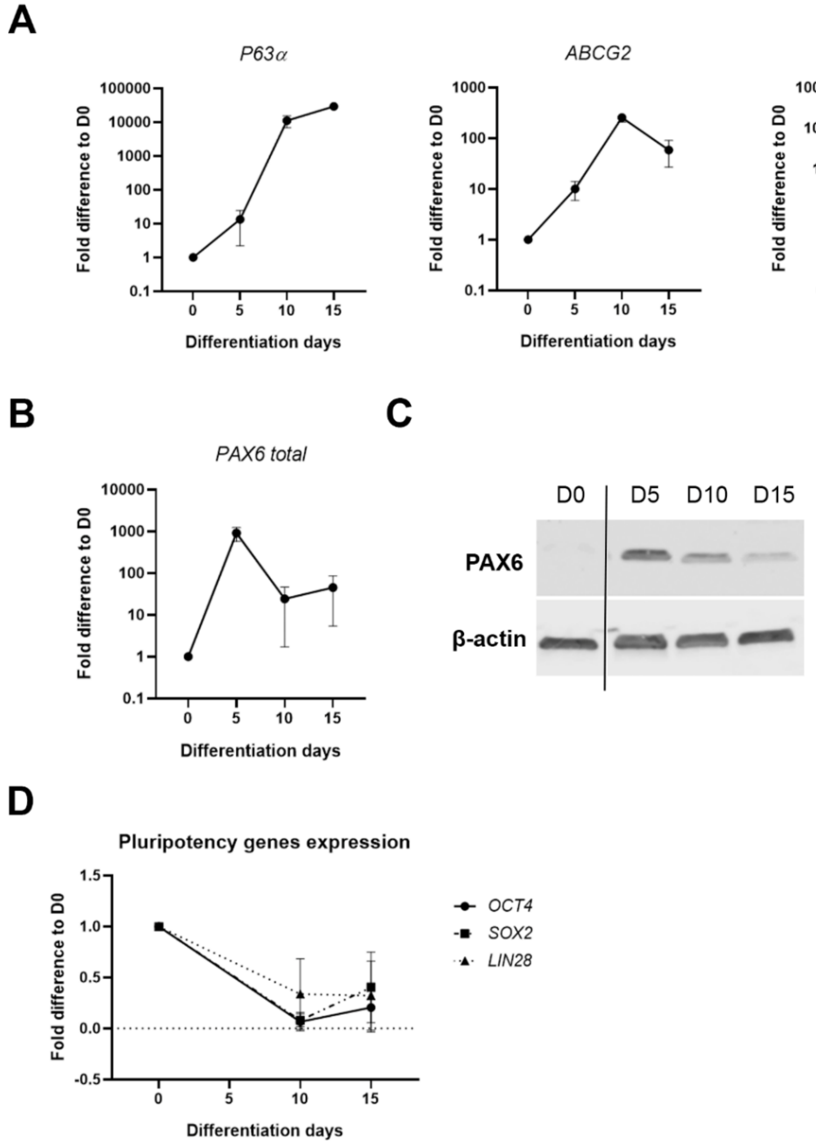

Figure S4. H9 embryonic stem cell (ESC) differentiation into Limbal epithelial stem cells (LESCs). (A) Expression of LESC specific markers *P63α, ABCG2* and *KRT14* was upregulated by day 15 and comparable to other lines shown in Figure 2. (B) The same pattern of expression of *PAX6* was also shown in this cell line. Values were normalised to day 0 and to internal housekeeper gene *GAPDH* (n=2). (C) Example of western blot showing PAX6 protein levels at different timepoints of LESC differentiation. (D) Pluripotency markers *OCT4*, *SOX2* and *LIN28* were downregulated through differentiation (n=2).

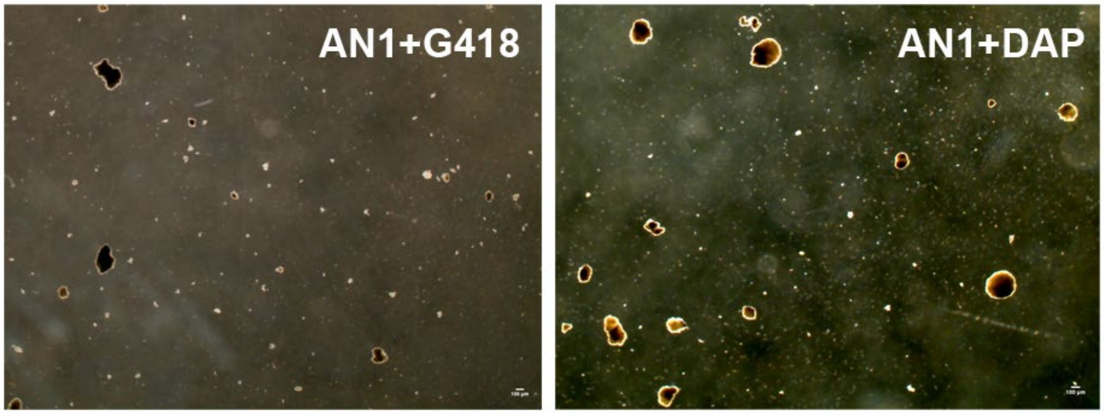

Figure S5. Cell toxicity after treatment of aniridia iPSC-derived optic cups with G418 100µg/mL and 2,6-diaminopurine (DAP) 200µM. Cell clumps progressively darkened after starting of dosing (day 15) and no viable structures were seen after day 20 (G418) or 25 (DAP) of treatment. Scale bar, 100µm.

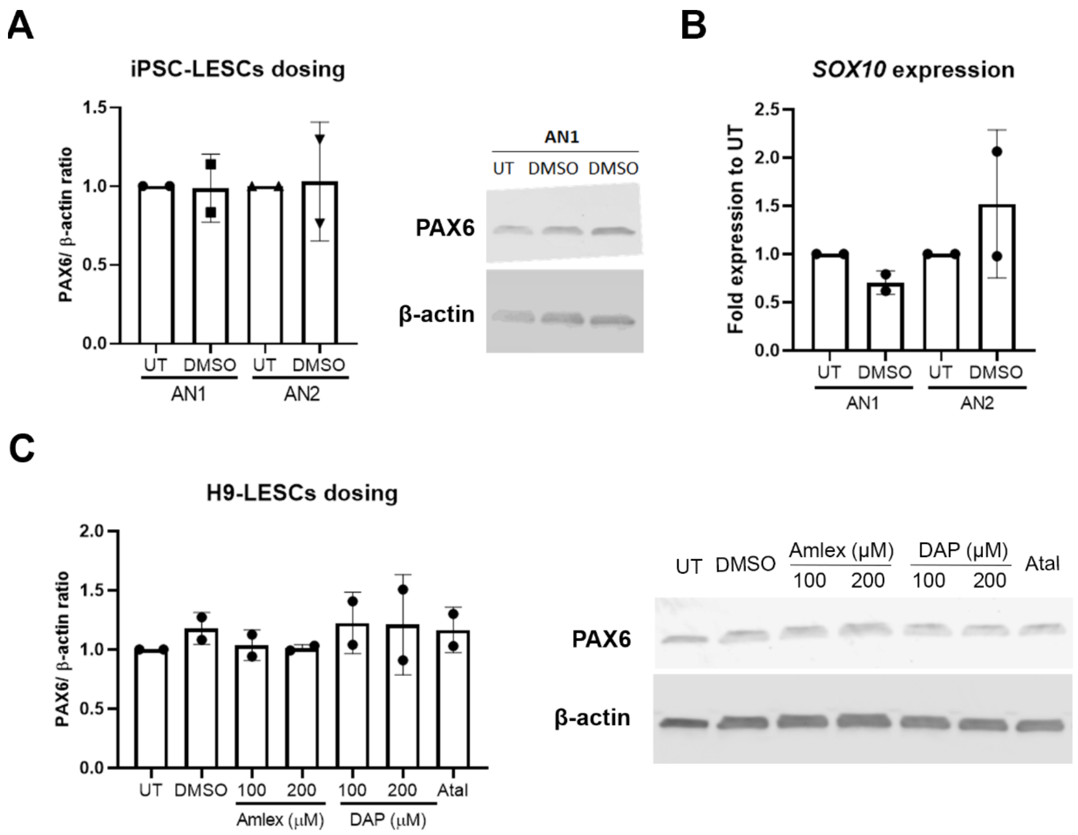

Figure S6. TRIDs dosing of aniridia and control (H9) iPSC-LESCs. (A) No significant changes found in PAX6 protein levels in AN1 and AN2 iPSC-LESCs untreated (UT) or after 48h dosing with vehicle (DMSO) treatment (n=2). (B) *SOX10* expression detected by qRT-PCR was not significantly different between UT and DMSO-treated AN1 and AN2 iPSC-LESCs (n=2). (C) Quantification of PAX6 protein in control H9 ESC-derived LESCs treated with different TRIDs. PAX6/β-actin ratio was normalised to untreated H9-LESCs. No significant differences between the different conditions were detected (n=2).

Table S1. Primer sequences used for qRT-PCR.

Marker	Forward sequence (5'-3')	Reverse sequence (5'-3')	Reference
GAPDH	ACA GTT GCC ATG TAG ACC	TTT TTG GTT GAG CAC AGG	In house
ACTB	TTC TAC AAT GAG CTG CGT G	GGG GTG TTG AAG GTC TCA AA	In house
PAX6	GGC CGA ACA GAC ACA GCC CTC AC	ATC ATA ACT CCG CCC ATT CAC C	In house
RAX	AGG CGG AAA AAT AGA GTT TG	TAC CCC AAT ATT CAC TCC TC	KickStart, Sigma Aldrich
VSX2	GGC GAC ACA GGA CAA TCT TTA	TTC CGG CAG CTC CGT TTT C	KickStart, Sigma Aldrich
MKi67	AAA CCA ACA AAG AGG AAC ACA AAT T	GTC TGG AGC GCA GGG ATA TTC	In house
TP63α	ATG TCG AAA TTG CTC AGG GAT TTT CAG A	TGA CCA CCA TCT ATC AGA TTG AGC ATT ACT	Foster et al, 2019
ΔNP63	GAA AAC AAT GCC CAG ACT CAA TTT	TCT GCG CGT GGT CTG TGT TAT	Foster et al, 2019
ABCG2	TCC ACT GCT GTG GCA TTA AA	CCT GCT TGG AAG GCT CTA TG	Foster et al, 2019
KRT14	CGG CCT GCT GAG ATC AAA GA	TCT GCA GAA GGA CAT TGG CA	Foster et al, 2019

SOX10	CTC TGG AGG CTG CTG AA	TGG GCT GGT ACT TGT AGT C	Leung et al, 2016
